# Supplementary material for: Self-report and device-based physical activity measures and adherence to physical activity recommendations: a cross-sectional survey among people with inflammatory joint disease in four European countries
Source: BMJ Open. 2023 Feb 6;13(2):e064278. doi: 10.1136/bmjopen-2022-064278 (PMC9906173; doi:10.1136/bmjopen-2022-064278)
Supplement: Supplementary data [file bmjopen-2022-064278supp001.pdf]

Supplementary file – Survey

A survey of people with inflammatory joint diseases use of physical activity measures

Demographics

* 1. Gender

- Male
- Female

* 2. What is your age?

- 18 to 24
- 25 to 34
- 35 to 44
- 45 to 54
- 55 to 64
- 65 to 74
- 75 or older

* 3. What type of arthritis have you been diagnosed with?

- Rheumatoid Arthritis (RA)
- Ankylosing Spondylitis (AS)
- Psoriatic Arthritis (PsA)

Other (please specify)

* 4. What year did you first experience your symptoms?

* 5. What body region do you have pain in?

- [ ] Arm
- [ ] Hand
- [ ] Foot
- [ ] Leg
- [ ] Spine
- [ ] Neck
- [ ] Head
- [ ] Other

Other (please specify)

6. Does your health limit you in the following activities?

	Yes, limited a lot	Yes, limited a little	No, not limited at all
Moderate activities e.g. moving a table, vacuum cleaning, bowling, golf etc.	☐	☐	☐
Climbing several flights of stairs	☐	☐	☐

* 7. What type of medication are you on?

- ◯ I do not take medications
- ◯ Biological DMARDS
- ◯ Non-biological DMARDS
- ◯ I do not know

Other (please specify)

* 8. What is your working situation?

- ◯ Full-time employment
- ◯ Part-time employment
- ◯ Unemployed
- ◯ Retired (due to age)
- ◯ Disability
- ◯ Housewife/Househusband

Other (please specify)

* 9. What is your highest level of education?

- ◯ Primary level
- ◯ Secondary level
- ◯ Third level

Other (please specify)

Views on measuring Physical Activity

* 10. Do you think it is important to measure physical activity?

- ☐ Yes
- ☐ No

11. Why do you/don't you think it is important to measure physical activity?

12. Where do you find your information on measuring physical activity?

- [ ] I don't
- [ ] Physiotherapist
- [ ] Rheumatologist
- [ ] Occupational Therapist
- [ ] Nurse
- [ ] Friends
- [ ] GP/Family Doctor
- [ ] Internet
- [ ] Magazine/Newspaper

Other (please specify)

Physical Activity Measurement - Objective measurement

Physical Activity is important for people with IJD's. While the measurement and monitoring of same can be a challenge, it has a number of essential uses. The following questions are concerned with the more popular ways in which Physical Activity is monitored and your answers will guide the evidence base and practice of health promotion.

13. How familiar are you with the following devices/ways in monitoring physical activity? (0 is not familiar and 10 is very familiar)

	0	1	2	3	4	5	6	7	8	9	10
PEDOMETER 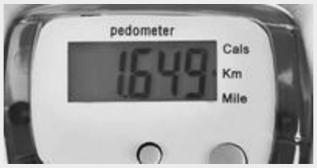	○	○	○	○	○	○	○	○	○	○	○
ACCELEROMETER 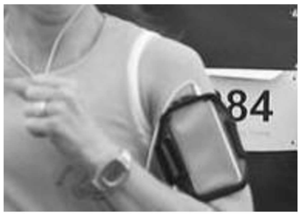	○	○	○	○	○	○	○	○	○	○	○
INTERNET 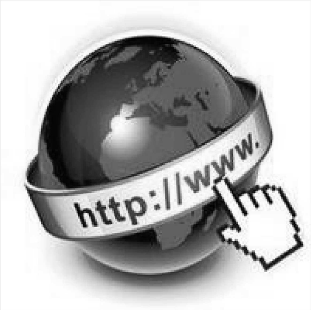	○	○	○	○	○	○	○	○	○	○	○
SMARTPHONE APP 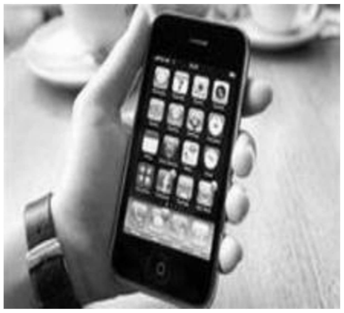	○	○	○	○	○	○	○	○	○	○	○

	0	1	2	3	4	5	6	7	8	9	10
QUESTIONNAIRE 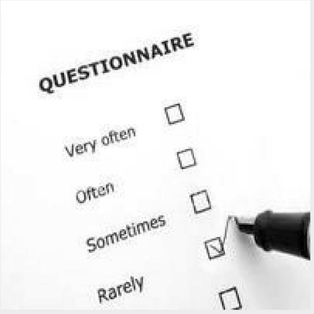	◯	◯	◯	◯	◯	◯	◯	◯	◯	◯	◯
DIARY 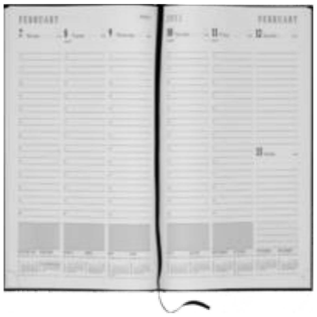	◯	◯	◯	◯	◯	◯	◯	◯	◯	◯	◯

14. The following questions are in relation to PEDOMETERS

How familiar are you in using a Pedometer to measure Physical Activity?

◯ Very familiar

◯ Somewhat familiar

◯ Vaguely familiar

◯ Not familiar

15. Do you own a Pedometer?

◯ Yes

◯ No

16. If Yes, where did you get the Pedometer?

- ◯ I bought it
- ◯ A gift
- ◯ Provided by my employer/work place
- ◯ From a study
- ◯ From a Health Professional

Other (please specify)

17. If Yes, how frequently do you wear a Pedometer?

- ◯ Every Day
- ◯ Most Days
- ◯ Rarely wear it
- ◯ Never wear it

18. Have you been instructed in how to use a Pedometer by a Health Professional within Rheumatology?

- ◯ Yes
- ◯ No

19. If Yes, who has instructed you in using it?

- ◯ Nurse
- ◯ Rheumatologist
- ◯ Physiotherapist
- ◯ Occupational Therapist

Other (please specify)

20. How familiar are you in reading the results from a Pedometer?

- Very familiar
- Somewhat familiar
- Vaguely familiar
- Not familiar

21. The following questions are in relation to ACCELEROMETERS

How familiar are you in using an Accelerometer for measuring Physical Activity?

- Very familiar
- Somewhat familiar
- Vaguely familiar
- Not familiar

22. Do you own an Accelerometer?

- Yes
- No

23. If Yes, where did you get the Accelerometer?

- I bought it
- A gift
- Provided by my employer/work place
- From a study
- From a Health Professional

Other (please specify)

24. If Yes, how frequently do you wear an Accelerometer?

- Every Day
- Most Days
- Rarely wear it
- Never wear it

25. Have you been instructed in how to use an Accelerometer by a Health Professional within Rheumatology?

- Yes
- No

26. If Yes, who has instructed you in using it?

- Nurse
- Rheumatologist
- Physiotherapist
- Occupational Therapist

Other (please specify)

27. How familiar are you in reading the results from an Accelerometer?

- Very familiar
- Somewhat familiar
- Vaguely familiar
- Not familiar

28. The following questions are in relation to the INTERNET

How familiar are you in using the INTERNET for measuring Physical Activity?

- Very familiar
- Somewhat familiar
- Vaguely familiar
- Not familiar

29. Do you use the INTERNET for measuring Physical Activity?

- Yes
- No

30. If Yes, have any of the following recommended you to use it?

- Nurse
- Rheumatologist
- Physiotherapist
- Occupational Therapist

Other (please specify)

31. If Yes, how frequently do you use the INTERNET to measure your Physical Activity?

- Every Day
- Most Days
- Rarely wear it
- Never wear it

32. Have you been instructed in how to use the INTERENET to measure your Physical Activity by a Health Professional within Rheumatology?

- Yes
- No

33. If Yes, who has instructed you in using it?

- Nurse
- Rheumatologist
- Physiotherapist
- Occupational Therapist

Other (please specify)

34. How familiar are you in reading the results of your Physical Activity from the INTERNET?

- ◯ Very familiar
- ◯ Somewhat familiar
- ◯ Vaguely familiar
- ◯ Not familiar

35. The following questions are in relation to SMARTPHONE APPS

How familiar are you in using a Smartphone App for measuring Physical Activity?

- ◯ Very familiar
- ◯ Somewhat familiar
- ◯ Vaguely familiar
- ◯ Not familiar

36. Do you have a Smartphone App for Physical Activity?

- ◯ Yes
- ◯ No

37. If Yes, where did you get your Smartphone App?

- ◯ I downloaded it - Free
- ◯ I downloaded it - Paid
- ◯ A gift
- ◯ Provided by my employer/work place
- ◯ From a study
- ◯ From a Health Professional

Other (please specify)

38. If Yes, how frequently do you use a Smartphone App?

- ◯ Every Day
- ◯ Most Days
- ◯ Rarely wear it
- ◯ Never wear it

39. Have you been instructed in how to use a Smartphone App by a Health Professional within Rheumatology?

- ◯ Yes
- ◯ No

40. If Yes, who has instructed you in using it?

- ◯ Nurse
- ◯ Rheumatologist
- ◯ Physiotherapist
- ◯ Occupational Therapist

Other (please specify)

41. How familiar are you in reading the results from a Smartphone App?

- Very familiar
- Somewhat familiar
- Vaguely familiar
- Not familiar

42. The following questions are in relation to QUESTIONNAIRES

How familiar are you in using a Questionnaire or Diary for measuring Physical Activity?

- Very familiar
- Somewhat familiar
- Vaguely familiar
- Not familiar

43. Do you use a Questionnaire for measuring Physical Activity?

- Yes
- No

44. If Yes, have any of the following recommended you to use it?

- Nurse
- Rheumatologist
- Physiotherapist
- Occupational Therapist

Other (please specify)

45. If Yes, how frequently do you use a Questionnaire to measure your Physical Activity?

- Every Day
- Most Days
- Rarely wear it
- Never wear it

46. How familiar are you in reading the results of your Physical Activity from a Questionnaire?

- Very familiar
- Somewhat familiar
- Vaguely familiar
- Not familiar

47. The following questions are in relation to DIARIES

How familiar are you in using a Diary for measuring Physical Activity?

- Very familiar
- Somewhat familiar
- Vaguely familiar
- Not familiar

48. Do you use a Diary for measuring Physical Activity?

- Yes
- No

49. If Yes, have any of the following recommended you to use it?

- Nurse
- Rheumatologist
- Physiotherapist
- Occupational Therapist

Other (please specify)

50. If Yes, how frequently do you use a Diary to measure your Physical Activity?

- Every Day
- Most Days
- Rarely wear it
- Never wear it

51. How familiar are you in reading the results of your Physical Activity from a Diary?

- Very familiar
- Somewhat familiar
- Vaguely familiar
- Not familiar

Barriers and Preferences

52. Do you think there are any barriers to people with arthritis measuring their own physical activity levels?

- Yes
- No

53. If Yes, please give an example

54. In your opinion, what is the best way to measure Physical Activity for people with arthritis?

* Pedometer
* Accelerometer
* Internet
* Smartphone app
* Questionnaire
* Diary

Current Physical Activity Levels

* 55. Think about an average week in the past few months and please answer the following regarding yourown Physical activites

	How many days per week?	Average time in minutes per day?	Intensity of activity
Commuting Activities i.e. Walk, Cycle etc.			
Work Activities i.e. Walking, Standing, Lifting etc.			
Leisure Time Activities i.e. Walking, Cycling, Gardening etc.			
Sport Activities i.e. Tennis, Football, Rugby etc.			
Household Activities i.e. Cleaning, Washing etc.			
