# Supplementary material for: Self-report and device-based physical activity measures and adherence to physical activity recommendations: a cross-sectional survey among people with inflammatory joint disease in four European countries
Source: BMJ Open. 2023 Feb 6;13(2):e064278. doi: 10.1136/bmjopen-2022-064278 (PMC9906173; doi:10.1136/bmjopen-2022-064278)
Supplement: Author's manuscript [file bmjopen-2022-064278.draft_revisions.pdf]

BMJ Open

BMJ Open is committed to open peer review. As part of this commitment we make the peer review history of every article we publish publicly available.

When an article is published we post the peer reviewers' comments and the authors' responses online. We also post the versions of the paper that were used during peer review. These are the versions that the peer review comments apply to.

The versions of the paper that follow are the versions that were submitted during the peer review process. They are not the versions of record or the final published versions. They should not be cited or distributed as the published version of this manuscript.

BMJ Open is an open access journal and the full, final, typeset and author-corrected version of record of the manuscript is available on our site with no access controls, subscription charges or pay-per-view fees (http://bmjopen.bmj.com).

If you have any questions on BMJ Open's open peer review process please email info.bmjopen@bmj.com

BMJ Open

Self-report and device-based physical activity measures and their contribution to adherence to physical activity recommendations. A cross-sectional survey among people with inflammatory joint disease in four European countries.

Journal:	*BMJ Open*
Manuscript ID	bmjopen-2022-064278
Article Type:	Original research
Date Submitted by the Author:	29-Apr-2022

Complete List of Authors:	Brodin, N; Karolinska Institutet, Department of Neurobiology Care Sciences and Society, Division of Physiotherapy; Danderyds Sjukhus AB, Division of Physiotherapy, Orthopaedic Clinic<br>Conradsson, David; Karolinska Institutet Department of Neurobiology Care Sciences and Society, Division of Physiotherapy; Karolinska University Hospital, Medical unit Occupational therapy & Physiotherapy, Theme Women's Health and Allied Health Professional<br>Swinnen, Thijs; KU Leuven, Department of Rehabilitation Sciences; KU Leuven University Hospitals Leuven, Division of Rheumatology<br>Appel Esbensen, Bente; Rigshospitalet; University of Copenhagen, Department of Clinical Medicine<br>Kennedy, Norelee; University of Limerick, Discipline of Physiotherapy, School of Allied Health; University of Limerick, Health Research Institute,<br>Hammer, Nanna Maria ; Rigshospitalet, Copenhagen Center for Arthritis Research (Copecare)<br>McKenna, Sean; University of Limerick, Discipline of Physiotherapy, School of Allied Health; University of Limerick Hospitals Group, Health Service Executive<br>Henriksson, Peter ; Karolinska Institutet, Department of clinical sciences, Danderyds hospital<br>Nordgren, Birgitta; Karolinska Institutet Department of Neurobiology Care Sciences and Society, Division of Physiotherapy; Karolinska University Hospital, Medical unit Occupational therapy & Physiotherapy, Theme Women's Health and Allied Health Professional

Keywords:	Musculoskeletal disorders < ORTHOPAEDIC & TRAUMA SURGERY, Rheumatology < INTERNAL MEDICINE, Health informatics < BIOTECHNOLOGY & BIOINFORMATICS

SCHOLARONE™
Manuscripts

I, the Submitting Author has the right to grant and does grant on behalf of all authors of the Work (as defined in the below author licence), an exclusive licence and/or a non-exclusive licence for contributions from authors who are: i) UK Crown employees; ii) where BMJ has agreed a CC-BY licence shall apply, and/or iii) in accordance with the terms applicable for US Federal Government officers or employees acting as part of their official duties; on a worldwide, perpetual, irrevocable, royalty-free basis to BMJ Publishing Group Ltd ("BMJ") its licensees and where the relevant Journal is co-owned by BMJ to the co-owners of the Journal, to publish the Work in this journal and any other BMJ products and to exploit all rights, as set out in our licence.

The Submitting Author accepts and understands that any supply made under these terms is made by BMJ to the Submitting Author unless you are acting as an employee on behalf of your employer or a postgraduate student of an affiliated institution which is paying any applicable article publishing charge ("APC") for Open Access articles. Where the Submitting Author wishes to make the Work available on an Open Access basis (and intends to pay the relevant APC), the terms of reuse of such Open Access shall be governed by a Creative Commons licence – details of these licences and which Creative Commons licence will apply to this Work are set out in our licence referred to above.

Other than as permitted in any relevant BMJ Author's Self Archiving Policies, I confirm this Work has not been accepted for publication elsewhere, is not being considered for publication elsewhere and does not duplicate material already published. I confirm all authors consent to publication of this Work and authorise the granting of this licence.

For peer review only

Self-report and device-based physical activity measures and their contribution to adherence to physical activity recommendations. A cross-sectional survey among people with inflammatory joint disease in four European countries.

Brodin N[1,2] Moulaee Conradsson D [1,3], Swinnen TWS [4,5], Esbensen B.A [6,7], Kennedy N [8,9], Hammer NM[6], McKenna S[8,10], Henriksson P[11], Nordgren B[1,3]

Authors affiliation

1 Division of Physiotherapy, Department of Neurobiology, Care Sciences and Society, Karolinska Institutet, Stockholm, Sweden

2 Division of Physiotherapy, Orthopaedic Clinic, Danderyds Sjukhus AB, Stockholm, Sweden

3 Medical unit Occupational therapy & Physiotherapy, Theme Women's Health and Allied Health Professional, Karolinska University Hospital, Stockholm, Sweden

4 Department of Rehabilitation Sciences, Research Group for Musculoskeletal Rehabilitation, KU Leuven, Leuven, Belgium

5 Division of Rheumatology, UZ Leuven, Leuven, Belgium

6 Copenhagen Center for Arthritis Research, Center for Rheumatology and Spine Diseases, Centre of Head and Orthopaedics, Rigshospitalet, Glostrup, Denmark

7 Department of Clinical Medicine, University of Copenhagen, Copenhagen, Denmark

8 Discipline of Physiotherapy, School of Allied Health, University of Limerick, Limerick, Ireland

9 Health Research Institute, University of Limerick, Limerick, Ireland

10 Health Service Executive, University Hospitals Limerick Group, Limerick

11 Department of Clinical Sciences Danderyd Hospital, Karolinska Institutet, Stockholm, Sweden

Corresponding Author

Dr Birgitta Nordgren

Division of Physiotherapy, Department of Neurobiology, Care Sciences and Society, Karolinska Institutet, Stockholm, Sweden

For peer review only - http://bmjopen.bmj.com/site/about/guidelines.xhtml

Medical unit Occupational therapy & Physiotherapy, Theme Women's Health and Allied Health Professional, Karolinska University Hospital, Stockholm, Sweden

birgitta.nordgren@ki.se

For peer review only

ABSTRACT

Objectives: Self-monitoring of physical activity (PA) has the potential to track PA behaviour and seems to contribute to successful behaviour change in PA interventions in different populations, including people with inflammatory joint diseases (IJDs). The objectives of this study were to describe the use, knowledge and sources of information of self-report- and device-based PA measures in people with IJDs in four European countries, and to explore if the use of such devices, socio-demographic factors or disease-related variables contribute to adherence to the recommendations of at least 150 minutes of moderate to vigorous PA per week.

Setting: Cross-sectional survey

Participants: People with IJDs in four European countries, i.e., Belgium, Denmark, Ireland and Sweden.

Primary and secondary outcome measures: Use of self-report and device-based PA measures, receiving instructions how to use PA measures, confidence in using them, adherence to PA recommendations and associated factors for adherence to PA recommendations.

Results: Of 1,382 respondents, 1,305 answered questions on PA measures and of those 600 (46%) reported that they used any kind of self-report or device-based measures to self-monitor PA. A between country difference of 34% to 58% was observed. Twenty six of the 428 (6%) users of simple devices received instructions from health professional on how to use the device and of those 182 using the complex device, eight (4%) did. Using a PA device was an independent associated factor of fulfilment of recommendations of PA (OR = 1,71, P< .001).

Conclusions: There is good use of technology among people with IJDs to measure their own PA signaling the value of such devices in improving overall PA awareness and engagement. PA self-monitoring was associated with meeting public PA health guidelines in a real-life setting.

For peer review only - http://bmjopen.bmj.com/site/about/guidelines.xhtml

Strengths and limitations of the study

- First study describing the use of self-report and/or device-based PA measures in ipeople with IJD in a real-world setting across four European countries
- An online questionnaire was used and distributed through patient organizations channels to reach a large part of the target population
- Due to the cross-sectional design, conclusions on causal relationships cannot be drawn

INTRODUCTION

Regular physical activity (PA) is associated with lower risk of cardiovascular disease, diabetes and several cancers. Healthy behaviours such as sufficient PA is a challenge for most people but may be even more challenging for people with inflammatory joint diseases (IJDs), as IJDs are characterized by functional limitations, pain and fatigue (1, 2).

PA defined as "any bodily movement produced by skeletal muscles that result in energy expenditure" (3) is safe and can improve disease activity, pain, fatigue, quality of life, and sleep in people with IJDs (4, 5). Despite these beneficial health outcomes, people with IJDs have lower PA levels than their healthier counterparts and many do not meet the required PA recommendations (6-10).

Self-monitoring of PA has the potential to track PA behaviour and seems to contribute to successful behaviour change in PA interventions in different populations, including people with IJDs (11-14). Physical activity can be monitored in different ways, although measurement properties (i.e., validity, reliability and responsiveness to change) vary considerably across techniques. Common methods for monitoring PA range from simple questionnaires and

For peer review only - http://bmjopen.bmj.com/site/about/guidelines.xhtml

pedometers to more complex devices such as accelerometers (15). While self-reported measures such as diaries and questionnaires as well as device-based PA measures can raise awareness and motivate patients to initiate and maintain PA, especially device-based measures show potential to mirror actual PA behaviour (14). Moreover, health professionals (HP) play an important role in supporting PA behavior in people with IJDs and could contribute to better health outcomes through promoting PA monitoring in clinical practice (16-18). Better knowledge and understanding of people with IJDs use and awareness of PA measures and its association to PA levels is needed to guide future practice of health promotion in this population. The use of PA measures in different European countries and to what extent people with IJDs perceive it to be important in measuring PA behaviour is in its infancy and needs to be further explored.

The main objective of this study was therefore to describe the use, knowledge and sources of information of self-report- and device-based PA measures in people with IJD in four European countries. A further objective was to explore if the use of such devices, socio-demographic factors or disease-related variables contribute to adherence to the recommendations of at least 150 minutes of moderate to vigorous PA per week.

METHODS

Design

Cross-sectional design using questionnaire was chosen as it allowed for the collection of data from a wider range of participants. The STROBE reporting guidelines for observational studies were used to guide the reporting of this study (19).

Patient and Public Involvement

For peer review only - http://bmjopen.bmj.com/site/about/guidelines.xhtml

Patients representing different IJDs and from all four countries were involved in the design of the questionnaire. This was organized following the same structure in all countries to identify missing or problematic questions/constructs in the questionnaire. All input was considered in the final questionnaire.

Sample

People with IJDs in four European countries, i.e., Belgium, Denmark, Ireland and Sweden were invited to participate in the study. In Belgium, people were identified through an outpatient university clinic and through patient organisations. In Denmark and Ireland, people were identified through the country's patient organisation membership. In Sweden, recruitment was performed through a Facebook group hosted by the patient organisation and from an outpatient rheumatology clinic. The chairperson for each country's patient organisation was contacted, requesting permission for their members to participate in the study. Following the granted permission, the chairpersons in each country received information via email, containing study information, survey link and researcher details and they provided the members with the same. In Sweden, participants recruited at the rheumatology clinic received the same information mentioned above as well as the paper questionnaire distributed through the patient organisation. Consent to participate was provided by responding to the survey anonymously. Detailed information on the study was provided on the first page of the survey. Separate ethical approvals were approved by each participating country's relevant research ethics committees (Belgium: S58849, Denmark: H-15013386, Ireland: 2015_09_02_EHS, Sweden: 2015/1785-31/5).

Questionnaire

The study steering group developed a questionnaire divided into four domains: 1) sociodemographic; 2) disease-related factors, 3) PA self-monitoring measures; questionnaire

and diary (paper or digital), simple body worn PA devices, (e.g., simple wearable activity trackers), or complex body worn devices (e.g., accelerometer, and wearable activity trackers with wireless link to smartphone app or website) and 4) questions about the importance of measuring PA, confidence in using PA measures; and if having been recommended by an HP to self-monitor PA using any self-report or device-derived measure (online supplementary file).

The questionnaire was developed in English and translated into each country's language. To ascertain face validity of each country's version of the questionnaire, discussions were organised with health professionals' i.e., Physiotherapist, Occupational Therapist and a Clinical Nurse Specialist. In addition, interviews with four people diagnosed with different IJD (Rheumatoid Arthritis (RA) n=2, Ankylosing Spondylitis (AS) n=1 and Psoriatic Arthritis (PSA) n=1)) to cover different disease spectra were also organised country-wise. These discussions took place to explore whether the constructs surveyed within each questionnaire reflected the aims under study (i.e., to identify missing or problematic questions/constructs). We therefore applied a purposive sampling method to reflect different diagnoses of arthritis, age, and gender and subsequently the questionnaires were adapted to improve their readability, validity and specificity. The final questionnaires were piloted with people with IJD and experts in this field to ensure content validity. Thus, verifying whether questions were readable, relevant and representative of the study's aims, in addition to minimise the risk of missing data. PA levels were measured using the The Short Questionnaire to Assess Health Enhancing Physical Activity (SQUASH) questionnaire (20). The SQUASH allows a comparison of national and international PA recommendations and includes questions on commuting activities (walking, cycling), PA at work or school, household activities and leisure time activities (walking, gardening, cycling, sports). The participants were asked to refer to a normal week during the last month; how many days a week they engaged in each of the

For peer review only - http://bmjopen.bmj.com/site/about/guidelines.xhtml

activities, the average time per day spent on each activity (hours and minutes) and the effort of each activity (low, moderate, high). Activities were assigned a Metabolic Equivalent Task value (MET) according to Ainsworth's' compendium of physical activities (21). Based on reported effort of the activities in the questionnaire, activities were assigned an intensity score between 1-9, were ≥3 assumed to represent health-enhancing PA. Study participants, who reported 150 min or more of moderate or more intensive activities per week, were classified as reaching health-enhancing PA recommendations. This was calculated by summing up the number of days per week for activities on a moderate or higher intensity level where the total duration was 30 minutes or more. A minimum of five days resulted in patients being categorized as reaching the health-enhancing PA recommendations (5). The SQUASH has previously demonstrated good test–retest reliability and modest construct validity in the IJD population (22).

Data Collection

The survey was conducted online during 2015-2016 with one reminder sent. For participants in Belgium, Denmark, and Ireland the survey was distributed through SurveyMonkey or SurveyXact. In Sweden the survey was conducted online through Artologik Survey&Report as well as through paper questionnaire distributed at an outpatient rheumatology clinic.

Analysis

Statistical analyses were carried out using IBM SPSS, version 27.0 (SPSS Inc., Chicago, Illinois, USA).

For the main objective, the results were presented descriptively using proportions and percentage or median and inter quartile range (IQR) for categorical variables and means and standard deviations (SD) for continuous variables.

For peer review only - http://bmjopen.bmj.com/site/about/guidelines.xhtml

For further objectives, Table 1 display variables and criteria used for categorization of independent and dependent variables for logistic regressions.

Table 1. Variables and criteria used for categorization of the independent variables and the dependent variable.

**Variables and instruments**	**Criteria for categorization**
Independent variables	
Age	^{3}55 years/<55 years
Sex	Female/male
Work status	Working/not working
Education	Higher/basic
Bio-DMARDs medication	Treatment/No treatment
Non-biol medication	Treatment/No treatment
Activity limitation - moderate activities	No limitation/Limitations
Activity limitation -stairs	No limitation/Limitations
Pain arm	No pain/pain
Pain hand	No pain/pain
Pain foot	No pain/pain
Pain leg	No pain/pain
Think it is important to monitor physical activity	Yes/No
Use device measuring physical activity	Yes/No
Dependent variable	
≥150 minutes a week of moderate –high intensity physical activity	Yes/No

Firstly, potential associated factors for fulfilment of PA recommendation were entered one at a time in unadjusted univariate logistic regression models. At this point, the cut-off to include factors in the multivariate analyses was set to an alpha level P≤ 0.1. Prior to multivariable analyses, measures of association, i.e., Chi Square tests, were computed to assess for

For peer review only - http://bmjopen.bmj.com/site/about/guidelines.xhtml

For peer review only

collinearity among potential associated factors. Associated factors were subsequently entered into a multivariable logistic regression model with an alpha level set at P≤ 0.05 for the identification of independent associated factors. Results for logistic regressions are expressed as odds ratios (OR) and 95% confidence intervals (95% CI).

RESULTS

Study participants

A total of 1,382 participants with IJD in Belgium, Denmark, Ireland and Sweden answered the survey mainly online. A majority of the participants were diagnosed with RA (n = 554, 40%), 331 (24%) with AS, 296 (21%) with PSA and 201 (15%) with other diagnoses (Table 2).

Table 2 Demographic and disease-related factors and fulfilment of physical activity recommendations of participants

Variables	Belgium (n=591)	Denmark (n=579)	Ireland (n=90)	Sweden (n=122)	Total (n=1382)	Comparison between countries
Diagnosis n (%)						
RA	217 (37)	237 (41)	56 (62)	44 (36)	554 (40)	<0.001
AS	221 (37)	77 (13)	13 (14)	20 (16)	331(24)	
Female sex n (%)	409 (69)	509 (88)	69 (77)	108 (86)	1095 (79)	<0.001
Age, < 55years (%)	263 (45)	347 (60)	48 (53)	48 (39)	706 (51)	<0.001
Years symptom, mean (SD)	17.9 (12.4)	13.7 (10.8)	10.1 (7.9)	15.2	15.4 (11.8)	<0.001
Bio-DMARDs n (%)	150 (25)	136 (24)	39 (43)	48 (40)	373 (27)	<0.001
Non-biol DMARDs n (%)	105 (18)	306 (53)	26 (29)	39 (32)	476 (34)	<0.001

For peer review only - http://bmjopen.bmj.com/site/about/guidelines.xhtml

Basic education n (%)	289 (49)	259 (45)	37 (41)	60 (49)	645 (48)	0.595
Working full/part time n (%)	203 (34)	234 (40)	44 (49)	70 (57)	551 (40)	<0.001
Pain						
Hand n (%)	285 (48)	371 (64)	40 (44)	81 (66)	777 (56)	<0.001
Arm n (%)	149 (25)	327 (57)	50 (56)	79 (65)	605 (44)	<0.001
Foot n (%)	248 (42)	306 (53)	31 (34)	69 (57)	654 (47)	<0.001
Leg n (%)	135 (23)	335 (58)	43 (48)	95 (79)	608 (44)	<0.001
Back n (%)	323 (55)	307 (53)	18 (20)	66 (54)	714 (52)	<0.001
Neck n (%)	271 (46)	219 (38)	15 (17)	49 (40)	554 (40)	<0.001
Head n (%)	80 (14)	55 (9)	2 (2)	12 (9)	149 (11)	0.006
Activity limitation						
Limited a lot	113 (19)	156 (27)	21 (23)	17 (14)	307 (22)	0.057
moderate activities n (%)						
Limited a lot	60 (10)	147 (25)	26 (29)	20 (16)	253 (18)	<0.001
climbing stairs n (%)						
Own or use PA device n (%)	340 (58)	274 (47)	49 (54)	42 (34)	600 (43)	<0.001
Fulfilment of PA recommend	84 (14)	277 (48)	68 (76)	74 (61)	503 (36)	<0.001

RA: Rheumatodi arthritis, AS: Ankylosing spondylitis, PSA: Psoriatic arthritis, SD: Standard deviation, PA: Physical activity,
*other = fibromyalgia, lupus, systemic lupus erythematosus, Wegeners granulomatosis, psoriasis, scleroderma, arthritis

For peer review only

Descriptive data on demographic and disease-related factors and fulfilment of physical activity recommendations of participants is displayed in Table 2.

Use of PA measures

Of the included 1,382 participants, 1,305 answered questions on PA measures. Of those, 600 (46%) reported that they used any kind of self-report or device-based measures to self-monitor PA. The most common used measure was the simple body worn device, used by 428 (33%), whereof 112 used it regularly. The complex body worn device were used by 182 (14%), whereof 56 used it regularly. One hundred and seven of 1,305 (8%) reported using digital or paper questionnaire and 104 (8%) used digital or paper diaries for self-monitoring of PA. Divided per country, the use of any kind of self-report or device-based measure differed between countries from 34% to 58% (Table 2).

Instructions of PA measures by HPs

Twenty-six of the 428 (6%) users of simple devices received instructions from HPs on how to use the device and of those 182 using the complex device, eight (4%) did. Health professionals instructed 52 (50%) of those 107 using digital/paper questionnaire and 10 out of 104 (10%) of those using diaries.

Perceived importance and confidence in self-monitoring of PA

All the 1,382 answered questions about importance and confidence. Most of the participants, 925 (67%) perceived it was important to measure PA and 558 (40%) reported they did not receive information about PA from any source. Of those receiving information about PA, 388 (28%) received information from a physiotherapist, 363 (26%) from a rheumatologist, 299 (22%) from internet, 159 (12%) from a general practitioner and 147 (11%) from newspapers or

For peer review only - http://bmjopen.bmj.com/site/about/guidelines.xhtml

magazines. A small number of the study participants received information from friends, 59 (4%), from an occupational therapist 46 (3%) or from a nurse 38 (3%).

The participants were most confident in using and interpreting PA data from simple body worn devices and paper questionnaires. They were least confident in using complex body worn sensors collecting multiple data, as well as using digital diaries (Table 3).

Table 3. Participants confidence/familiarity in using self-reports and devices based physical activity measures (n = 1305)

	n	Median (IQR)
Simple (0-10)*	1305	3 (8)
Complex sensor (0-10)	1305	1 (6)
Paper QR (0-10)	1305	3 (5)
Paper diary (0-10)	1304	1 (5)
Digital QR (0-10)	1305	1 (5)
Digital diary (0-10)	1305	0 (5)

* possible score range 0-10 0= not confident, 10= very confident
IQR= interquartile range, QR= questionnaire

Fulfilment of physical activity recommendations and its associated factors

A total of 503 of the 1,382 (36%) participants reported they fulfilled the recommendations of ≥ 150 minutes of PA on a moderate intensity per week, whereof 418 (39%) were female.

Univariate logistic regression analyses (Table 4) revealed that people with IJD who were <55 years of age (OR = 1.46, P= .001), working part- or fulltime (OR = 1.61, P< .001), being treated with non-biological disease modifying medication (OR = 1.54, P< .001), with no perceived activity limitations in moderate activities (OR = 1.41, P< .001) and who were using a device to measure PA (OR = 1.93, P< .001) were more likely to fulfil the recommendations of PA. In contrast, those of male sex (OR = 0.68, P= .007) and not experiencing pain in the arm (OR =

For peer review only - http://bmjopen.bmj.com/site/about/guidelines.xhtml

0.56, P< .001) or leg (OR = 0.65, P< .001) were less likely to fulfil the recommendations of PA.

Table 4. Univariate and multiple logistic regression models for factors associated with fulfilment of recommendations of physical activity.

Variable	*Univariate regression*		*Multiple regression*	
	OR (95% CI)	*P-value*	OR (95% CI)	*P-value*
Age				
<55 years	1.46 (1.74–1.83)	**.001**	1.19 (0.94–1.52)	.152
≥55 years	1.00 (reference)		1.00 (reference)	
Sex				
Male	0.68 (0.51–0.90)	**.007**	0.81 (0.59–1.09)	.167
Female	1.00 (reference)		1.00 (reference)	
Work status				
Working	1.61 (1.28–2.01)	**<.001**	1.38 (1.08–1.76)	**.001**
Not working	1.00 (reference)		1.00 (reference)	
Education				
Higher education	0.89 (0.81–1.27)	.895		
Basic education	1.00 (reference)			
Bio-DMARDs medication				
Treatment	1.24 (0.97–1.60)	.084		
No treatment	1.00 (reference)			
Non-biol medication				
Treatment	1.54 (1.22–1.94)	**<.001**	1.18 (0.92–1.51)	.185
No treatment	1.00 (reference)		1.00 (reference)	
Activity limitation – moderate activities				
No limitation	1.41 (1.09–1.83)	**<.001**	1.76 (1.31–2.36)	**<.001**
Limitations	1.00 (reference)		1.00 (reference)	
Activity limitation – climbing stairs				
No limitation	1.00 (0.79–1.27)	.990		
Limitations	1.00 (reference)			
Pain arm				
No pain	0.56 (0.44–0.69)	**<.001**	0.56 (0.44–0.73)	**<.001**
Pain	1.00 (reference)		1.00 (reference)	
Pain hand				
No pain	0.89 (0.72–1.12)	.328		
Pain	1.00 (reference)			
Pain foot				
No pain	1.10 (0.88–1.37)	.407		
Pain	1.00 (reference)			

For peer review only

Pain leg				
No pain	0.65 (0.52–0.81)	**<.001**	0.79 (0.61–1.01)	.068
Pain	1.00 (reference)		1.00 (reference)	
Think it is important to monitor physical activity				
Yes	1.26 (0.99–1.61)	.058		
No	1.00 (reference)			
Use device to measure physical activity				
Yes	1.93 (1.53–2.42)	**<.001**	1.71 (1.35–2.16)	**<.001**
No	1.00 (reference)		1.00 (reference)	
OR: odds ratio;				

The multivariable logistic regression model (Table 4) showed that working part- or fulltime (OR = 1.38, P= .001), not perceiving activity limitations in moderate activities (OR = 1.76, P< .001), not experiencing arm pain (OR = 0.56, P< .001) and using a device to measure PA (OR = 1,71, P< .001) remained independent associated factors of fulfilment of recommendations of PA. In this model, 64.1% of the outcome was correctly classified.

DISCUSSION

To the best of our knowledge this is the first study describing the use of self-report and/or device-based PA measures in individuals with IJD in a real-world situation, across four European countries and its association to fulfilment of PA recommendations.

Overall, close to 50% of the participants used any kind of self-report or device-based measures to self-monitor PA. Furthermore, in our cohort 37% used device-based measures, which is comparable to the general population (23). This is promising given the motivation for PA which comes with the use of devices and also their advantages of simplicity and accuracy (24-25). A between country difference of 34% to 58% was observed. It can be considered obvious that younger participants to a larger extent would be those who use wearable trackers. This could be considered supported by the data from Sweden, which has

For peer review only - http://bmjopen.bmj.com/site/about/guidelines.xhtml

the lowest amount of tracker users, and also is the country with the fewest participants under the age of 55 years. However, Belgium had the highest number of tracker users although the country did not have the youngest group. Hence, other explanations to the between country differences may be sought beyond the variables measured in this study.

Only 112 (8%) of the participants used simple body worn devices and 56 (4%) used complex body worn devices regularly. This is in line with previous interventions aiming to increase PA using wearable trackers in people with rheumatic- and musculoskeletal diseases, showing that adherence to use of trackers over longer periods of time is not maintained (25).

Although a majority (67%) of our sample perceived it was important to measure PA, 40% did not receive information from a HP on why it is important, which is similar to an earlier study in an arthritis population (18). Of those using simple and complex devices only 6% and 4% respectively received instructions on how to use the devices. On the other hand, a substantial part, 50%, reported they were instructed how to use questionnaires to measure PA. One explanation to this could be that HPs are used to using traditional questionnaires in clinic and therefore might be more prone to inform their patients about them. The factor most strongly associated with engaging in PA is getting recommendation from a HP and previous research has identified low HP knowledge about PA and exercise as a reason for not guiding patients with IJD in PA (26). Even though HPs are aware of benefits and barriers in relation to PA in IJD, promotion and advice is conflicted by HPs lack of certainty with respect to joint damage and fear of flare-up (27). Moreover, recent studies highlight further educational needs and development for HPs to support PA in this population (16, 28). While PA devices can give the user real-time feedback of daily steps or energy expenditure, patients with IJDs would most likely benefit from also adding HPs support providing them with tailored feed-back to

For peer review only - http://bmjopen.bmj.com/site/about/guidelines.xhtml

motivate PA (1). In fact, better long-term adherence has been shown in interventions incorporating feedback and behavioural change techniques compared with activity devices alone (29).

Using a PA device was an independent associated factor in our multivariate logistic regression model of adherence to PA recommendations also when considering sociodemographic- and disease-related factors, support from HPs, and participants own perception of familiarity and confidence of using PA devices. As this is a cross-sectional study, we cannot determine if the measure itself motivates to PA in this sample, or if the more physically active participants use the measures for self-monitoring of their behaviour. However, it clearly highlights that using PA devices is one of the contributors to higher/increased PA in this and other populations and such should thus be taken into account in the promotion of PA (25, 30, 31). Other factors associated to adherence to PA recommendations in our sample were working part- or full time, not perceiving activity limitations as well as not having pain in arms. These factors provide information for clinicians to identify which patients need more targeted support to improve their PA levels, an important consideration in managing busy clinical loads.

Limitations

First, the study had a cross-sectional design therefore, conclusions on causal relationships cannot be drawn. This could have been helped by a follow up over time to allow for firmer conclusions about causality.

Second, the questionnaire was based on self-report through social networking, which may have attracted socially desired answers. This is an inherent limitation in most surveys and while it was ensured the data collection was anonymous, self-reports are never free from bias (32). Moreover, most of those who answered were those involved in the patient organization,

For peer review only - http://bmjopen.bmj.com/site/about/guidelines.xhtml

probably with a specific interest in their arthritis, and most likely had a particular interest in the topic. However, as inclusion also took place through outpatient clinics in both Belgium and Sweden, our sample is probably representative of a broader range of characteristics as the patient organizations' members are generally older, thus not working and not on disability leave as they are retired. It should be noted that the overall response rate could not be calculated, as exact numbers of members of the patient organizations were not available or could not be retrieved. Another point which could be regarded as a limitation is that the recruitment procedure through the patient organizations was not similar in each country. In Belgium, Denmark and Ireland invitations to participate were sent via email to members of the patient organizations while in Sweden recruitment took place through the Swedish Rheumatism Association's Facebook page.

As reported earlier in our previous study, investigating rheumatology HPs awareness of PA measures in people with IJDs (28) we cannot neglect the risk of respondents having misinterpreted questions in the survey. Simultaneously with the survey used in the prior study (28) the present survey was designed in English and translated into Swedish, Danish, French and Flemish with results back-translated into English for the reporting. Through the different translations, there is a risk that valuable information was lost. Face validity was investigated in each country before the survey was used, and part of the included questions were other validated questionnaires (20, 33).

CONCLUSION

Despite these limitations, this study contributes and provides insight into the use of PA measures and devices in a real-world setting by people with IJDs. This is important in order to enhance the collaborative promotion of PA between health professional and patient (29). The

For peer review only - http://bmjopen.bmj.com/site/about/guidelines.xhtml

study found that there is good use of technology among people with IJDs to measure their own PA signaling the value of such devices in improving overall PA awareness and engagement. Moreover, PA self-monitoring was associated with meeting public PA health guidelines in a real-life setting. The study also identified factors associated with PA device use allowing for more targeted management of people with IJDs to improve PA. It also highlights the need for HP engagement with PA advice and understanding of PA devices as an important part of the care of people with IJDs.

Acknowledgement

The authors thank Sara Wiljeminis who assisted in designing the survey and collecting the data.

Contributor ship statement

NB, BN, NK, BAE, and TWS were the original authors who submitted the grant, designed the study, oversaw the data collection and analysis in their countries, prepared the paper and read and commented on all drafts and agreed on the final manuscript. SGMcK, and NMH were research assistants who assisted with designing the survey, collecting the data, and commented on manuscript drafts. DMC and PH undertook the statistical analyses and commented on all manuscript drafts.

Competing interests

None declared

Funding

The study was funded by the European Leauge Against Rheumatism Health Professional Research Grant 2015.

Data Sharing Statement

For peer review only - http://bmjopen.bmj.com/site/about/guidelines.xhtml

No additional data available

Ethics Approval

This study was approved by the Medical Ethical Committee of the University Hospitals Leuven S58849 in Belgium, by the Ethics Committee of the Capital Region of Denmark H-15013386 in Denmark, by the University of Limerick research ethics committee: 2015_09_02_EHS in Ireland and by the Swedish Etikprövningsmyndigheten 2015/1785-31/5 in Sweden.

REFERENCES

1. Veldhuijzen van Zanten JJ, Rouse PC, Hale ED, Ntoumanis N, Metsios GS, Duda JL, et al. Perceived Barriers, Facilitators and Benefits for Regular Physical Activity and Exercise in Patients with Rheumatoid Arthritis: A Review of the Literature. Sports Med. 2015;45(10):1401-12.

2. Collado-Mateo D, Lavín-Pérez AM, Peñacoba C, Del Coso J, Leyton-Román M, Luque-Casado A, et al. Key Factors Associated with Adherence to Physical Exercise in Patients with Chronic Diseases and Older Adults: An Umbrella Review. Int J Environ Res Public Health. 2021;18(4).

3. Caspersen CJ, Powell KE, Christenson GM. Physical activity, exercise, and physical fitness: definitions and distinctions for health-related research. Public health reports (Washington, DC : 1974). 1985;100(2):126-31.

4. Durcan L, Wilson F, Cunnane G. The effect of exercise on sleep and fatigue in rheumatoid arthritis: a randomized controlled study. The Journal of rheumatology. 2014;41(10):1966-73.

5. Rausch Osthoff AK, Niedermann K, Braun J, Adams J, Brodin N, Dagfinrud H, et al. 2018 EULAR recommendations for physical activity in people with inflammatory arthritis and osteoarthritis. Ann Rheum Dis. 2018;77(9):1251-60.

6. Hootman JM, Macera CA, Ham SA, Helmick CG, Sniezek JE. Physical activity levels among the general US adult population and in adults with and without arthritis. Arthritis Rheum. 2003;49(1):129-35.

For peer review only - http://bmjopen.bmj.com/site/about/guidelines.xhtml

7. Tierney M, Fraser A, Kennedy N. Physical activity in rheumatoid arthritis: a systematic review. J Phys Act Health. 2012;9(7):1036-48.

8. Jacquemin C, Servy H, Molto A, Sellam J, Foltz V, Gandjbakhch F, et al. Physical Activity Assessment Using an Activity Tracker in Patients with Rheumatoid Arthritis and Axial Spondyloarthritis: Prospective Observational Study. JMIR mHealth and uHealth. 2018;6(1):e1.

9. Barker J, Smith Byrne K, Doherty A, Foster C, Rahimi K, Ramakrishnan R, et al. Physical activity of UK adults with chronic disease: cross-sectional analysis of accelerometer-measured physical activity in 96 706 UK Biobank participants. International journal of epidemiology. 2019;48(4):1167-74.

10. Haider S, Sedlak M, Kapan A, Grabovac I, Lamprecht T, Erlacher L, et al. Factors Associated with Objectively Measured Physical Activity in Patients with Seropositive Rheumatoid Arthritis. Int J Environ Res Public Health. 2020;17(23).

11. Michie S, Ashford S, Sniehotta FF, Dombrowski SU, Bishop A, French DP. A refined taxonomy of behaviour change techniques to help people change their physical activity and healthy eating behaviours: the CALO-RE taxonomy. Psychology & health. 2011;26(11):1479-98.

12. Page EJ, Massey AS, Prado-Romero PN, Albadawi S. The Use of Self-Monitoring and Technology to Increase Physical Activity: A Review of the Literature. Perspectives on behavior science. 2020;43(3):501-14.

13. Compernolle S, DeSmet A, Poppe L, Crombez G, De Bourdeaudhuij I, Cardon G, et al. Effectiveness of interventions using self-monitoring to reduce sedentary behavior in adults: a systematic review and meta-analysis. The international journal of behavioral nutrition and physical activity. 2019;16(1):63.

14. Patel MS, Asch DA, Volpp KG. Wearable devices as facilitators, not drivers, of health behavior change. Jama. 2015;313(5):459-60.

15. Dowd KP, Szeklicki R, Minetto MA, Murphy MH, Polito A, Ghigo E, et al. A systematic literature review of reviews on techniques for physical activity measurement in adults: a DEDIPAC study. The international journal of behavioral nutrition and physical activity. 2018;15(1):15.

16. Swärdh E, Opava C, Brodin N. Physical activity in patients with rheumatoid arthritis - an agile lifelong behaviour: a qualitative meta-synthesis. RMD open. 2021;7(2).

For peer review only - http://bmjopen.bmj.com/site/about/guidelines.xhtml

17. Freid LM, Ogdie A, Baker JF. Physical Activity Patterns in People With Inflammatory Arthritis Indicate They Have not Received Recommendation-Based Guidance From Health Care Providers. ACR open rheumatology. 2020;2(10):582-7.

18. Fontaine KR, Bartlett SJ, Heo M. Are health care professionals advising adults with arthritis to become more physically active? Arthritis Rheum. 2005;53(2):279-83.

19. Vandenbroucke JP, von Elm E, Altman DG, Gøtzsche PC, Mulrow CD, Pocock SJ, et al. Strengthening the Reporting of Observational Studies in Epidemiology (STROBE): explanation and elaboration. PLoS medicine. 2007;4(10):e297.

20. Wendel-Vos GC, Schuit AJ, Saris WH, Kromhout D. Reproducibility and relative validity of the short questionnaire to assess health-enhancing physical activity. Journal of clinical epidemiology. 2003;56(12):1163-9.

21. Ainsworth BE, Haskell WL, Leon AS, Jacobs DR, Jr., Montoye HJ, Sallis JF, et al. Compendium of physical activities: classification of energy costs of human physical activities. Medicine and science in sports and exercise. 1993;25(1):71-80.

22. Arends S, Hofman M, Kamsma YP, van der Veer E, Houtman PM, Kallenberg CG, et al. Daily physical activity in ankylosing spondylitis: validity and reliability of the IPAQ and SQUASH and the relation with clinical assessments. Arthritis Res Ther. 2013;15(4):R99.

23. Alley S, Schoeppe S, Guertler D, Jennings C, Duncan MJ, Vandelanotte C. Interest and preferences for using advanced physical activity tracking devices: results of a national cross-sectional survey. BMJ open. 2016;6(7):e011243.

24. Sylvia LG, Bernstein EE, Hubbard JL, Keating L, Anderson EJ. Practical guide to measuring physical activity. Journal of the Academy of Nutrition and Dietetics. 2014;114(2):199-208.

25. Davergne T, Pallot A, Dechartres A, Fautrel B, Gossec L. Use of Wearable Activity Trackers to Improve Physical Activity Behavior in Patients With Rheumatic and Musculoskeletal Diseases: A Systematic Review and Meta-Analysis. Arthritis Care Res (Hoboken). 2019;71(6):758-67.

26. O'Donnell S, Rusu C, Bernatsky S, Hawker G, Canizares M, Mackay C, et al. Exercise/physical activity and weight management efforts in Canadians with self-reported arthritis. Arthritis Care Res (Hoboken). 2013;65(12):2015-23.

For peer review only - http://bmjopen.bmj.com/site/about/guidelines.xhtml

27. Halls S, Law RJ, Jones JG, Markland DA, Maddison PJ, Thom JM. Health Professionals' Perceptions of the Effects of Exercise on Joint Health in Rheumatoid Arthritis Patients. Musculoskeletal care. 2017;15(3):196-209.

28. Kennedy NM, McKenna SG, O'Neill A, Appel Esbensen B, Swinnen TW, Nordgren B, et al. A survey across four European countries to determine rheumatology health professionals' awareness of physical activity measures in people with inflammatory joint diseases. BMJ open. 2018;8(5):e020809.

29. Brickwood KJ, Watson G, O'Brien J, Williams AD. Consumer-Based Wearable Activity Trackers Increase Physical Activity Participation: Systematic Review and Meta-Analysis. JMIR mHealth and uHealth. 2019;7(4):e11819.

30. Li C, Chen X, Bi X. Wearable activity trackers for promoting physical activity: A systematic meta-analytic review. International journal of medical informatics. 2021;152:104487.

31. Ashur C, Cascino TM, Lewis C, Townsend W, Sen A, Pekmezi D, et al. Do Wearable Activity Trackers Increase Physical Activity Among Cardiac Rehabilitation Participants? A SYSTEMATIC REVIEW AND META-ANALYSIS. Journal of cardiopulmonary rehabilitation and prevention. 2021;41(4):249-56.

32. van de Mortel TF. Faking it: social desirability response bias in self-report research. Australian Journal of Advanced Nursing. 2008;25(4):40-8.

33. Linton SJ, Halldén K. Can we screen for problematic back pain? A screening questionnaire for predicting outcome in acute and subacute back pain. The Clinical journal of pain. 1998;14(3):209-15.

.

For peer review only - http://bmjopen.bmj.com/site/about/guidelines.xhtml

For peer review only

For peer review only - http://bmjopen.bmj.com/site/about/guidelines.xhtml

Supplementary file – Survey

A survey of people with inflammatory joint diseases use of physical activity measures

Demographics

* 1. Gender

- Male
- Female

* 2. What is your age?

- 18 to 24
- 25 to 34
- 35 to 44
- 45 to 54
- 55 to 64
- 65 to 74
- 75 or older

* 3. What type of arthritis have you been diagnosed with?

- Rheumatoid Arthritis (RA)
- Ankylosing Spondylitis (AS)
- Psoriatic Arthritis (PsA)

Other (please specify)

* 4. What year did you first experience your symptoms?

For peer review only

* 5. What body region do you have pain in?

- [ ] Arm
- [ ] Hand
- [ ] Foot
- [ ] Leg
- [ ] Spine
- [ ] Neck
- [ ] Head
- [ ] Other

Other (please specify)

6. Does your health limit you in the following activities?

	Yes, limited a lot	Yes, limited a little	No, not limited at all
Moderate activities e.g. moving a table, vacuum cleaning, bowling, golf etc.	☐	☐	☐
Climbing several flights of stairs	☐	☐	☐

For peer review only

* 7. What type of medication are you on?

- ◯ I do not take medications
- ◯ Biological DMARDS
- ◯ Non-biological DMARDS
- ◯ I do not know

Other (please specify)

* 8. What is your working situation?

- ◯ Full-time employment
- ◯ Part-time employment
- ◯ Unemployed
- ◯ Retired (due to age)
- ◯ Disability
- ◯ Housewife/Househusband

Other (please specify)

* 9. What is your highest level of education?

- ◯ Primary level
- ◯ Secondary level
- ◯ Third level

Other (please specify)

Views on measuring Physical Activity

* 10. Do you think it is important to measure physical activity?

- ☐ Yes
- ☐ No

11. Why do you/don't you think it is important to measure physical activity?

For peer review only

12. Where do you find your information on measuring physical activity?

- [ ] I don't
- [ ] Physiotherapist
- [ ] Rheumatologist
- [ ] Occupational Therapist
- [ ] Nurse
- [ ] Friends
- [ ] GP/Family Doctor
- [ ] Internet
- [ ] Magazine/Newspaper

Other (please specify)

Physical Activity Measurement - Objective measurement

Physical Activity is important for people with IJD's. While the measurement and monitoring of same can be a challenge, it has a number of essential uses. The following questions are concerned with the more popular ways in which Physical Activity is monitored and your answers will guide the evidence base and practice of health promotion.

13. How familiar are you with the following devices/ways in monitoring physical activity? (0 is not familiar and 10 is very familiar)

For peer review only

	0	1	2	3	4	5	6	7	8	9	10
PEDOMETER	○	○	○	○	○	○	○	○	○	○	○
ACCELEROMETER	○	○	○	○	○	○	○	○	○	○	○
INTERNET	○	○	○	○	○	○	○	○	○	○	○
SMARTPHONE APP	○	○	○	○	○	○	○	○	○	○	○

PEDOMETER

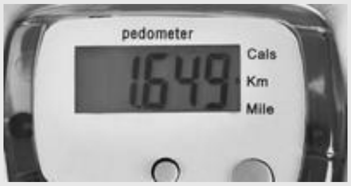

ACCELEROMETER

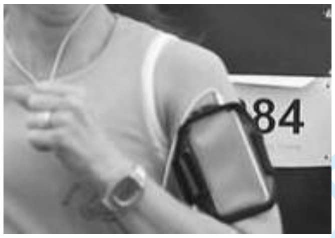

INTERNET

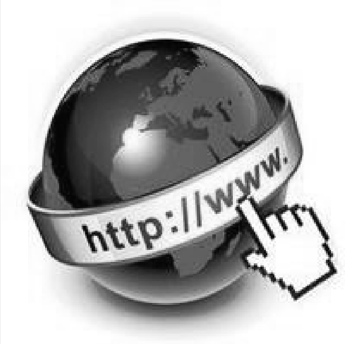

SMARTPHONE APP

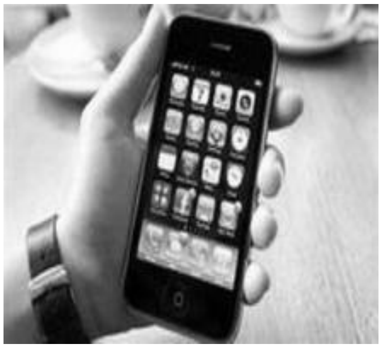

For peer review only

	0	1	2	3	4	5	6	7	8	9	10
QUESTIONNAIRE	○	○	○	○	○	○	○	○	○	○	○
DIARY	○	○	○	○	○	○	○	○	○	○	○

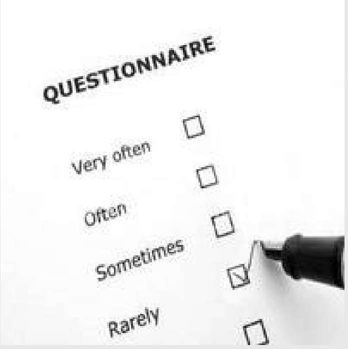

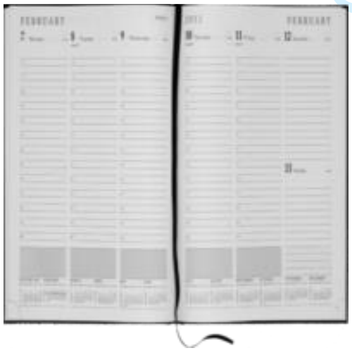

For peer review only

14. The following questions are in relation to PEDOMETERS

How familiar are you in using a Pedometer to measure Physical Activity?

- ○ Very familiar
- ○ Somewhat familiar
- ○ Vaguely familiar
- ○ Not familiar

15. Do you own a Pedometer?

- ○ Yes
- ○ No

16. If Yes, where did you get the Pedometer?

- I bought it
- A gift
- Provided by my employer/work place
- From a study
- From a Health Professional

Other (please specify)

17. If Yes, how frequently do you wear a Pedometer?

- Every Day
- Most Days
- Rarely wear it
- Never wear it

18. Have you been instructed in how to use a Pedometer by a Health Professional within Rheumatology?

- Yes
- No

19. If Yes, who has instructed you in using it?

- Nurse
- Rheumatologist
- Physiotherapist
- Occupational Therapist

Other (please specify)

For peer review only

20. How familiar are you in reading the results from a Pedometer?

- Very familiar
- Somewhat familiar
- Vaguely familiar
- Not familiar

21. The following questions are in relation to ACCELEROMETERS

How familiar are you in using an Accelerometer for measuring Physical Activity?

- Very familiar
- Somewhat familiar
- Vaguely familiar
- Not familiar

22. Do you own an Accelerometer?

- Yes
- No

23. If Yes, where did you get the Accelerometer?

- I bought it
- A gift
- Provided by my employer/work place
- From a study
- From a Health Professional

Other (please specify)

For peer review only

24. If Yes, how frequently do you wear an Accelerometer?

- Every Day
- Most Days
- Rarely wear it
- Never wear it

25. Have you been instructed in how to use an Accelerometer by a Health Professional within Rheumatology?

- Yes
- No

26. If Yes, who has instructed you in using it?

- Nurse
- Rheumatologist
- Physiotherapist
- Occupational Therapist

Other (please specify)

27. How familiar are you in reading the results from an Accelerometer?

- Very familiar
- Somewhat familiar
- Vaguely familiar
- Not familiar

28. The following questions are in relation to the INTERNET

How familiar are you in using the INTERNET for measuring Physical Activity?

- Very familiar
- Somewhat familiar
- Vaguely familiar
- Not familiar

For peer review only

29. Do you use the INTERNET for measuring Physical Activity?

- Yes
- No

30. If Yes, have any of the following recommended you to use it?

- Nurse
- Rheumatologist
- Physiotherapist
- Occupational Therapist

Other (please specify)

31. If Yes, how frequently do you use the INTERNET to measure your Physical Activity?

- Every Day
- Most Days
- Rarely wear it
- Never wear it

32. Have you been instructed in how to use the INTERENET to measure your Physical Activity by a Health Professional within Rheumatology?

- Yes
- No

33. If Yes, who has instructed you in using it?

- Nurse
- Rheumatologist
- Physiotherapist
- Occupational Therapist

Other (please specify)

For peer review only

34. How familiar are you in reading the results of your Physical Activity from the INTERNET?

- ◯ Very familiar
- ◯ Somewhat familiar
- ◯ Vaguely familiar
- ◯ Not familiar

35. The following questions are in relation to SMARTPHONE APPS

How familiar are you in using a Smartphone App for measuring Physical Activity?

- ◯ Very familiar
- ◯ Somewhat familiar
- ◯ Vaguely familiar
- ◯ Not familiar

36. Do you have a Smartphone App for Physical Activity?

- ◯ Yes
- ◯ No

For peer review only

37. If Yes, where did you get your Smartphone App?

- I downloaded it - Free
- I downloaded it - Paid
- A gift
- Provided by my employer/work place
- From a study
- From a Health Professional

Other (please specify)

38. If Yes, how frequently do you use a Smartphone App?

- Every Day
- Most Days
- Rarely wear it
- Never wear it

39. Have you been instructed in how to use a Smartphone App by a Health Professional within Rheumatology?

- Yes
- No

40. If Yes, who has instructed you in using it?

- Nurse
- Rheumatologist
- Physiotherapist
- Occupational Therapist

Other (please specify)

For peer review only

41. How familiar are you in reading the results from a Smartphone App?

- Very familiar
- Somewhat familiar
- Vaguely familiar
- Not familiar

42. The following questions are in relation to QUESTIONNAIRES

How familiar are you in using a Questionnaire or Diary for measuring Physical Activity?

- Very familiar
- Somewhat familiar
- Vaguely familiar
- Not familiar

43. Do you use a Questionnaire for measuring Physical Activity?

- Yes
- No

44. If Yes, have any of the following recommended you to use it?

- Nurse
- Rheumatologist
- Physiotherapist
- Occupational Therapist

Other (please specify)

For peer review only

45. If Yes, how frequently do you use a Questionnaire to measure your Physical Activity?

- Every Day
- Most Days
- Rarely wear it
- Never wear it

46. How familiar are you in reading the results of your Physical Activity from a Questionnaire?

- Very familiar
- Somewhat familiar
- Vaguely familiar
- Not familiar

47. The following questions are in relation to DIARIES

How familiar are you in using a Diary for measuring Physical Activity?

- Very familiar
- Somewhat familiar
- Vaguely familiar
- Not familiar

48. Do you use a Diary for measuring Physical Activity?

- Yes
- No

49. If Yes, have any of the following recommended you to use it?

- Nurse
- Rheumatologist
- Physiotherapist
- Occupational Therapist

Other (please specify)

For peer review only

50. If Yes, how frequently do you use a Diary to measure your Physical Activity?

- Every Day
- Most Days
- Rarely wear it
- Never wear it

51. How familiar are you in reading the results of your Physical Activity from a Diary?

- Very familiar
- Somewhat familiar
- Vaguely familiar
- Not familiar

Barriers and Preferences

52. Do you think there are any barriers to people with arthritis measuring their own physical activity levels?

- Yes
- No

53. If Yes, please give an example

54. In your opinion, what is the best way to measure Physical Activity for people with arthritis?

* Pedometer
* Accelerometer
* Internet
* Smartphone app
* Questionnaire
* Diary

For peer review only

Current Physical Activity Levels

* 55. Think about an average week in the past few months and please answer the following regarding yourown Physical activites

	How many days per week?	Average time in minutes per day?	Intensity of activity
Commuting Activities i.e. Walk, Cycle etc.			
Work Activities i.e. Walking, Standing, Lifting etc.			
Leisure Time Activities i.e. Walking, Cycling, Gardening etc.			
Sport Activities i.e. Tennis, Football, Rugby etc.			
Household Activities i.e. Cleaning, Washing etc.			

For peer review only

STROBE 2007 (v4) Statement—Checklist of items that should be included in reports of *cross-sectional studies*

Section/Topic	Item #	Recommendation	Reported on page #
**Title and abstract**	1	(*a*) Indicate the study's design with a commonly used term in the title or the abstract	1-3
		(*b*) Provide in the abstract an informative and balanced summary of what was done and what was found	2
**Introduction**			
Background/rationale	2	Explain the scientific background and rationale for the investigation being reported	3-4
Objectives	3	State specific objectives, including any prespecified hypotheses	4
**Methods**			
Study design	4	Present key elements of study design early in the paper	5
Setting	5	Describe the setting, locations, and relevant dates, including periods of recruitment, exposure, follow-up, and data collection	5-7
Participants	6	(*a*) Give the eligibility criteria, and the sources and methods of selection of participants	5
Variables	7	Clearly define all outcomes, exposures, predictors, potential confounders, and effect modifiers. Give diagnostic criteria, if applicable	5-6
Data sources/ measurement	8*	For each variable of interest, give sources of data and details of methods of assessment (measurement). Describe comparability of assessment methods if there is more than one group	5-8
Bias	9	Describe any efforts to address potential sources of bias	Supplementary file
Study size	10	Explain how the study size was arrived at	-
Quantitative variables	11	Explain how quantitative variables were handled in the analyses. If applicable, describe which groupings were chosen and why	8
Statistical methods	12	(*a*) Describe all statistical methods, including those used to control for confounding	8
		(*b*) Describe any methods used to examine subgroups and interactions	8
		(*c*) Explain how missing data were addressed	-
		(*d*) If applicable, describe analytical methods taking account of sampling strategy	-
		(*e*) Describe any sensitivity analyses	-
**Results**			

For peer review only

Participants	13*	(a) Report numbers of individuals at each stage of study—eg numbers potentially eligible, examined for eligibility, confirmed eligible, included in the study, completing follow-up, and analysed	8-9
		(b) Give reasons for non-participation at each stage	-
		(c) Consider use of a flow diagram	-
Descriptive data	14*	(a) Give characteristics of study participants (eg demographic, clinical, social) and information on exposures and potential confounders	Table 2
		(b) Indicate number of participants with missing data for each variable of interest	8-9, Table 2
Outcome data	15*	Report numbers of outcome events or summary measures	
Main results	16	(*a*) Give unadjusted estimates and, if applicable, confounder-adjusted estimates and their precision (eg, 95% confidence interval). Make clear which confounders were adjusted for and why they were included	Table 5
		(*b*) Report category boundaries when continuous variables were categorized	Table 1
		(*c*) If relevant, consider translating estimates of relative risk into absolute risk for a meaningful time period	Not applicable
Other analyses	17	Report other analyses done—eg analyses of subgroups and interactions, and sensitivity analyses	-
**Discussion**			
Key results	18	Summarise key results with reference to study objectives	10-11
Limitations	19	Discuss limitations of the study, taking into account sources of potential bias or imprecision. Discuss both direction and magnitude of any potential bias	13
Interpretation	20	Give a cautious overall interpretation of results considering objectives, limitations, multiplicity of analyses, results from similar studies, and other relevant evidence	10-12 + 14
Generalisability	21	Discuss the generalisability (external validity) of the study results	12, 13
**Other information**			
Funding	22	Give the source of funding and the role of the funders for the present study and, if applicable, for the original study on which the present article is based	15

*Give information separately for cases and controls in case-control studies and, if applicable, for exposed and unexposed groups in cohort and cross-sectional studies.

Note: An Explanation and Elaboration article discusses each checklist item and gives methodological background and published examples of transparent reporting. The STROBE checklist is best used in conjunction with this article (freely available on the Web sites of PLoS Medicine at http://www.plosmedicine.org/, Annals of Internal Medicine at http://www.annals.org/, and Epidemiology at http://www.epidem.com/). Information on the STROBE Initiative is available at www.strobe-statement.org.

For peer review only

Self-report and device-based physical activity measures and adherence to physical activity recommendations. A cross-sectional survey among people with inflammatory joint disease in four European countries.

Journal:	*BMJ Open*
Manuscript ID	bmjopen-2022-064278.R1
Article Type:	Original research
Date Submitted by the Author:	16-Nov-2022
Complete List of Authors:	Brodin, N; Karolinska Institutet, Department of Neurobiology Care Sciences and Society, Division of Physiotherapy; Danderyds Sjukhus AB, Division of Physiotherapy, Orthopaedic Clinic<br>Conradsson, David; Karolinska Institutet Department of Neurobiology Care Sciences and Society, Division of Physiotherapy; Karolinska University Hospital, Medical unit Occupational therapy & Physiotherapy, Theme Women's Health and Allied Health Professional<br>Swinnen, Thijs; KU Leuven, Department of Rehabilitation Sciences; KU Leuven University Hospitals Leuven, Division of Rheumatology<br>Esbensen, Bente; Rigshospitalet; University of Copenhagen, Department of Clinical Medicine<br>Kennedy, Norelee; University of Limerick, Discipline of Physiotherapy, School of Allied Health; University of Limerick, Health Research Institute,<br>Hammer, Nanna Maria ; Rigshospitalet, Copenhagen Center for Arthritis Research (Copecare)<br>McKenna, Sean; University of Limerick, Discipline of Physiotherapy, School of Allied Health; University of Limerick Hospitals Group, Health Service Executive<br>Henriksson, Peter ; Karolinska Institutet, Department of clinical sciences, Danderyds hospital<br>Nordgren, Birgitta; Karolinska Institutet Department of Neurobiology Care Sciences and Society, Division of Physiotherapy; Karolinska University Hospital, Medical unit Occupational therapy & Physiotherapy, Theme Women's Health and Allied Health Professional
**Primary Subject Heading**:	Rheumatology
Secondary Subject Heading:	Evidence based practice
Keywords:	Musculoskeletal disorders < ORTHOPAEDIC & TRAUMA SURGERY, Rheumatology < INTERNAL MEDICINE, Health informatics < BIOTECHNOLOGY & BIOINFORMATICS

I, the Submitting Author has the right to grant and does grant on behalf of all authors of the Work (as defined in the below author licence), an exclusive licence and/or a non-exclusive licence for contributions from authors who are: i) UK Crown employees; ii) where BMJ has agreed a CC-BY licence shall apply, and/or iii) in accordance with the terms applicable for US Federal Government officers or employees acting as part of their official duties; on a worldwide, perpetual, irrevocable, royalty-free basis to BMJ Publishing Group Ltd ("BMJ") its licensees and where the relevant Journal is co-owned by BMJ to the co-owners of the Journal, to publish the Work in this journal and any other BMJ products and to exploit all rights, as set out in our licence.

The Submitting Author accepts and understands that any supply made under these terms is made by BMJ to the Submitting Author unless you are acting as an employee on behalf of your employer or a postgraduate student of an affiliated institution which is paying any applicable article publishing charge ("APC") for Open Access articles. Where the Submitting Author wishes to make the Work available on an Open Access basis (and intends to pay the relevant APC), the terms of reuse of such Open Access shall be governed by a Creative Commons licence – details of these licences and which Creative Commons licence will apply to this Work are set out in our licence referred to above.

Other than as permitted in any relevant BMJ Author's Self Archiving Policies, I confirm this Work has not been accepted for publication elsewhere, is not being considered for publication elsewhere and does not duplicate material already published. I confirm all authors consent to publication of this Work and authorise the granting of this licence.

For peer review only

Self-report and device-based physical activity measures and adherence to physical activity recommendations. A cross-sectional survey among people with inflammatory joint disease in four European countries.

Brodin N[1,2] Moulaee Conradsson D [1,3], Swinnen TWS [4,5], Esbensen B.A [6,7], Kennedy N [8,9], Hammer NM[6], McKenna S[8,10], Henriksson P[11], Nordgren B[1,3]

Authors affiliation

1 Division of Physiotherapy, Department of Neurobiology, Care Sciences and Society, Karolinska Institutet, Stockholm, Sweden

2 Division of Physiotherapy, Orthopaedic Clinic, Danderyds Sjukhus Corp.,, Stockholm, Sweden

3 Medical unit Occupational therapy & Physiotherapy, Theme Women's Health and Allied Health Professional, Karolinska University Hospital, Stockholm, Sweden

4 Department of Rehabilitation Sciences, Research Group for Musculoskeletal Rehabilitation, KU Leuven, Leuven, Belgium

5 Division of Rheumatology, UZ Leuven, Leuven, Belgium

6 Copenhagen Center for Arthritis Research, Center for Rheumatology and Spine Diseases, Centre of Head and Orthopaedics, Rigshospitalet, Glostrup, Denmark

7 Department of Clinical Medicine, University of Copenhagen, Copenhagen, Denmark

8 Discipline of Physiotherapy, School of Allied Health, University of Limerick, Limerick, Ireland

9 Health Research Institute, University of Limerick, Limerick, Ireland

10 Health Service Executive, University Hospitals Limerick Group, Limerick, Ireland

11 Department of Clinical Sciences Danderyd Hospital, Karolinska Institutet, Stockholm, Sweden

Corresponding Author

Dr Birgitta Nordgren

Division of Physiotherapy, Department of Neurobiology, Care Sciences and Society, Karolinska Institutet, Stockholm, Sweden

For peer review only - http://bmjopen.bmj.com/site/about/guidelines.xhtml

Medical unit Occupational therapy & Physiotherapy, Theme Women's Health and Allied Health Professional, Karolinska University Hospital, Stockholm, Sweden

birgitta.nordgren@ki.se

For peer review only

ABSTRACT

Objectives: Self-monitoring of physical activity (PA) has the potential to contribute to successful behaviour change in PA interventions in different populations, including people with inflammatory joint diseases (IJDs). The objectives of this study were to describe the use and knowledge of self-report- and device-based PA measures in people with IJDs in four European countries, and to explore if the use of such devices, socio-demographic or disease-related variables were associated with adherence to the recommendations of at least 150 minutes of moderate to vigorous PA per week.

Setting: Cross-sectional survey, performed in 2015-2016.

Participants: People with IJDs in Belgium, Denmark, Ireland and Sweden.

Primary and secondary outcome measures: Use of self-report and device-based PA measures, receipt of instructions how to use PA measures, confidence in using them, adherence to PA recommendations and associated factors for adherence to PA recommendations.

Results: Of the 1, 305 respondents answering questions on PA measures, 600 (46%) reported use of any kind of self-report or device-based measures to self-monitor PA. Between country differences of 34% to 58% was observed. Six and four percent received instructions from health professionals on how to use simple and complex devices, respectively. Independent associated factors of fulfilment of recommendations of PA were living in Ireland (OR = 84.89, P< .001) and Sweden (OR = 1.68, P= .017) compared to living in Denmark, not perceiving activity limitations in moderate activities (OR = 1.92, P< .001) and using a device to measure PA (OR = 1.56, P< .001). Those living in Belgium (OR = 0.21, P< .001) were less likely to fulfil recommendations of PA.

Conclusions: There is good use of technology among people with IJDs to measure their own PA, although few use wearable devices regularly. The results indicate that participants meeting public PA health guidelines were engaged in self-monitoring of PA.

For peer review only - http://bmjopen.bmj.com/site/about/guidelines.xhtml

Strengths and limitations of the study

- First study describing the use of self-report and/or device-based PA measures in people with IJD in a real-world setting across four European countries
- Study participants had low confidence in using device-based PA measures
- Study participants might have been particularly interested in PA prompting their participation in the study, limiting external validity
- Due to the cross-sectional design, conclusions on causal relationships cannot be drawn

INTRODUCTION

Regular physical activity (PA) is associated with lower risk of cardiovascular disease, diabetes and several cancers. Healthy behaviours such as achieving sufficient PA is a challenge for most people but may be even more challenging for people with inflammatory joint diseases (IJDs), as IJDs are characterized by functional limitations, pain and fatigue (1, 2).

PA defined as "any bodily movement produced by skeletal muscles that result in energy expenditure" (3) is safe and can improve disease activity, pain, fatigue, quality of life, and sleep in people with IJDs (4, 5). Despite these beneficial health outcomes, people with IJDs have lower PA levels than their healthier counterparts and many do not meet the required PA recommendations (6-10).

Self-monitoring of PA has the potential to contribute to successful behaviour change in PA interventions in different populations, including people with IJDs (11-14). Physical activity can be monitored in different ways, although measurement properties (i.e., validity, reliability and

For peer review only - http://bmjopen.bmj.com/site/about/guidelines.xhtml

responsiveness to change) vary considerably across techniques. Common methods for monitoring PA range from simple questionnaires and pedometers to more complex devices such as accelerometers (15). Self-reported measures such as diaries and questionnaires as well as device-based PA measures can raise awareness and motivate patients to initiate and maintain PA (12). In particular, device-based wearable trackers can give the user valid data on, for example, daily steps or PA intensity levels (16). Given their superior feasibility, adequate reliability and sufficient construct validity in group analyses, self-reported PA questionnaires remain useful to relate PA behaviours to clinical outcome in larger arthritis research studies (17, 18). Moreover, health professionals (HP) play an important role in supporting PA behavior in people with IJDs and could contribute to better health outcomes through promoting PA monitoring in clinical practice (19-21). Better knowledge and understanding of people with IJDs use and awareness of PA measures and its association to PA levels is needed to guide future practice of health promotion in this population. The use of PA measures in different European countries and to what extent people with IJDs perceive it to be important in measuring PA behaviour needs to be further explored.

The main objective of this study was therefore to describe the use, knowledge and sources of information of self-report- and device-based PA measures in people with IJD in four European countries. A further objective was to explore if the use of such devices, socio-demographic factors or disease-related variables were associated with adherence to the recommendations of at least 150 minutes of moderate to vigorous PA per week.

METHODS

Design

Cross-sectional design using questionnaire was chosen as it allowed for the collection of data

For peer review only - http://bmjopen.bmj.com/site/about/guidelines.xhtml

from a wider range of participants. The STROBE reporting guidelines for observational studies were used to guide the reporting of this study (22).

Patient and Public Involvement

Patients representing different IJDs in the four countries were involved in the design of the questionnaire. This was organized following the same structure in all countries. The patients were invited by each country representative to give their views and input on the questionnaire. They were asked to comment on the content of the questionnaire and identify any missing or problematic questions/constructs. All input was considered in the final questionnaire.

Sample

People with IJDs in four European countries, i.e., Belgium, Denmark, Ireland and Sweden were invited to participate in the study. In Belgium, people were identified through an outpatient university clinic and through patient organisations. In Denmark and Ireland, people were identified through the country's patient organisation membership. In Sweden, recruitment was performed through a Facebook group hosted by the patient organisation and from an outpatient rheumatology clinic. The chairperson for each country's patient organisation was contacted, requesting permission for their members to participate in the study. Following the granted permission, the chairpersons in each country received information via email, containing study information, survey link and researcher details and they provided the members with the same. In Sweden, participants recruited at the rheumatology clinic received the same information mentioned above as well as the paper questionnaire distributed through the patient organisation. Consent to participate was provided by responding to the survey anonymously. Detailed information on the study was provided on the first page of the survey. Separate ethical approvals

For peer review only - http://bmjopen.bmj.com/site/about/guidelines.xhtml

were approved by each participating country's relevant research ethics committees (Belgium: S58849, Denmark: H-15013386, Ireland: 2015_09_02_EHS, Sweden: 2015/1785-31/5).

Questionnaire

The study steering group developed a questionnaire divided into four domains: 1) sociodemographic; 2) disease-related factors, 3) PA self-monitoring measures; questionnaire and diary (paper or digital), simple body worn PA devices, (e.g., simple wearable activity trackers), or complex body worn devices (e.g., accelerometer, and wearable activity trackers with wireless link to smartphone app or website) and 4) questions about receiving instructions how to use PA measures, the importance of measuring PA, confidence in using PA measures; and if having been recommended by an HP to self-monitor PA using any self-report or device-derived measure (online supplementary file).

The questionnaire was developed in English and translated into each country's language. To ascertain face validity of each country's version of the questionnaire, discussions were organised with health professionals' i.e., Physiotherapist, Occupational Therapist and a Clinical Nurse Specialist. In addition, interviews with four people diagnosed with different IJD (Rheumatoid Arthritis (RA) n=2, Ankylosing Spondylitis (AS) n=1 and Psoriatic Arthritis (PSA) n=1)) to cover different disease spectra were also organised country-wise. These discussions took place to explore whether the constructs surveyed within each questionnaire reflected the aims under study (i.e., to identify missing or problematic questions/constructs) and if the questions were well understood. We therefore applied a purposive sampling method to reflect different diagnoses of arthritis, age, and gender and subsequently the questionnaires were adapted to improve their readability, validity and specificity. The final questionnaires were piloted with people with IJD and experts in this field to ensure content validity. Thus, verifying

For peer review only - http://bmjopen.bmj.com/site/about/guidelines.xhtml

whether questions were readable, relevant and representative of the study's aims, in addition to minimise the risk of missing data.

PA levels were measured using the The Short Questionnaire to Assess Health Enhancing Physical Activity (SQUASH) questionnaire (23). The SQUASH allows a comparison of national and international PA recommendations and includes questions on commuting activities (walking, cycling), PA at work or school, household activities and leisure time activities (walking, gardening, cycling, sports). The participants were asked to refer to a normal week during the last month; how many days a week they engaged in each of the activities, the average time per day spent on each activity (hours and minutes) and the effort of each activity (low, moderate, high). Activities were assigned a Metabolic Equivalent Task value (MET) according to Ainsworth's' compendium of physical activities (24). Based on reported effort of the activities in the questionnaire, activities were assigned an intensity score between 1-9, were ≥ 3 assumed to represent health-enhancing PA. Study participants, who reported 150 min or more of moderate or more intensive activities per week, were classified as reaching health-enhancing PA recommendations. This was calculated by summing up the number of days per week for activities on a moderate or higher intensity level where the total duration was 30 minutes or more. A minimum of five days resulted in patients being categorized as reaching the health-enhancing PA recommendations (5). The SQUASH has previously demonstrated good test–retest reliability and modest construct validity in the IJD population (17).

Data Collection

The survey was conducted online during 2015-2016 with one reminder sent. For participants in Belgium, Denmark, and Ireland the survey was distributed through SurveyMonkey or

For peer review only - http://bmjopen.bmj.com/site/about/guidelines.xhtml

SurveyXact. In Sweden the survey was conducted online through Artologik Survey&Report as well as through paper questionnaire distributed at an outpatient rheumatology clinic.

Analysis

Statistical analyses were carried out using IBM SPSS, version 27.0 (SPSS Inc., Chicago, Illinois, USA).

For the main objective, the results were presented descriptively using proportions and percentage or median and inter quartile range (IQR) for categorical variables and means and standard deviations (SD) for continuous variables.

For further objectives, Table 1 display variables and criteria used for categorization of independent and dependent variables for logistic regressions.

Table 1. Variables and criteria used for categorization of the independent variables and the dependent variable.

Variables and instruments	Criteria for categorization
Independent variables	
Country	Belgium/ Denmark/Ireland/Sweden//
Age	≥55 years/<55 years
Sex	Female/male
Work status	Working/not working
Education[1]	Higher/basic
Bio-DMARDs medication	Treatment/No treatment
Non-biol medication	Treatment/No treatment
Activity limitation - moderate activities	No limitation/Limitations
Activity limitation -stairs	No limitation/Limitations
Pain arm	No pain/pain
Pain hand	No pain/pain

For peer review only - http://bmjopen.bmj.com/site/about/guidelines.xhtml

Pain foot	No pain/pain
Pain leg	No pain/pain
Think it is important to monitor physical activity	Yes/No
Use device measuring physical activity	Yes/No
Dependent variable	
≥150 minutes a week of moderate –high intensity physical activity	Yes/No

[1]Higher education = university level. Basic education = primary and secondary level

Firstly, potential associated factors for fulfilment of PA recommendation were entered one at a time in unadjusted univariate logistic regression models. At this point, the cut-off to include factors in the multivariate analyses was set to an alpha level P≤ 0.1. Prior to multivariable analyses, measures of association, i.e., Chi Square tests, were computed to assess for collinearity among potential associated factors. Associated factors were subsequently entered into a multivariable logistic regression model with an alpha level set at P≤ 0.05 for the identification of independent associated factors. Results for logistic regressions are expressed as odds ratios (OR) and 95% confidence intervals (95% CI).

RESULTS

Study participants

A total of 1,382 participants with IJD in Belgium, Denmark, Ireland and Sweden answered the survey mainly online. A majority of the participants were diagnosed with RA (n = 554, 40%), 331 (24%) with AS, 296 (21%) with PSA and 201 (15%) with other diagnoses (Table 2).

Table 2 Demographic and disease-related factors and fulfilment of physical activity recommendations of participants

For peer review only - http://bmjopen.bmj.com/site/about/guidelines.xhtml

Variables	**Belgium** (n=591)	**Denmark** (n=579)	**Ireland** (n=90)	**Sweden** (n=122)	**Total** (n=1382)
Diagnosis n (%)					
RA	217 (37)	237 (41)	56 (62)	44 (36)	554 (40)
AS	221 (37)	77 (13)	13 (14)	20 (16)	331(24)
PSA	101 (17)	162 (28)	15 (17)	18 (15)	296 (21)
Other[1]	52 (9)	103 (18)	6 (7)	40 (33)	201 (15)
Female sex n (%)	409 (69)	509 (88)	69 (77)	108 (86)	1095 (79)
Age, < 55years (%)	263 (45)	347 (60)	48 (53)	48 (39)	706 (51)
Years symptom, mean (SD)	17.9 (12.4)	13.7 (10.8)	10.1 (7.9)	15.2 (12.2)	15.4 (11.8)
Bio-DMARDs medication (%)	150 (25)	136 (24)	39 (43)	48 (40)	373 (27)
Non-biological medication n (%)	105 (18)	306 (53)	26 (29)	39 (32)	476 (34)
Basic education[2] n (%)	289 (49)	259 (45)	37 (41)	60 (49)	645 (48)
Working full/part time n (%)	203 (34)	234 (40)	44 (49)	70 (57)	551 (40)
Pain					
Hand n (%)	285 (48)	371 (64)	40 (44)	81 (66)	777 (56)
Arm n (%)	149 (25)	327 (57)	50 (56)	79 (65)	605 (44)
Foot n (%)	248 (42)	306 (53)	31 (34)	69 (57)	654 (47)
Leg n (%)	135 (23)	335 (58)	43 (48)	95 (79)	608 (44)
Back n (%)	323 (55)	307 (53)	18 (20)	66 (54)	714 (52)
Neck n (%)	271 (46)	219 (38)	15 (17)	49 (40)	554 (40)
Head n (%)	80 (14)	55 (9)	2 (2)	12 (9)	149 (11)
Activity limitation					
Limited a lot moderate activities n (%)	113 (19)	156 (27)	21 (23)	17 (14)	307 (22)
Limited a lot climbing stairs n (%)	60 (10)	147 (25)	26 (29)	20 (16)	253 (18)
Own or use PA device n (%)	340 (58)	274 (47)	49 (54)	42 (34)	600 (43)
Fulfilment of PA recommendations n (%)	84 (14)	277 (48)	68 (76)	74 (61)	503 (36)

RA: Rheumatoid arthritis, AS: Ankylosing spondylitis, PSA: Psoriatic arthritis, SD: Standard deviation, PA: Physical activity,
[1]other = fibromyalgia, lupus, systemic lupus erythematosus, Wegeners granulomatosis, psoriasis, scleroderma, arthritis
[2]Basic education = primary and secondary level

For peer review only - http://bmjopen.bmj.com/site/about/guidelines.xhtml

Descriptive data on demographic and disease-related factors and fulfilment of physical activity recommendations of participants is displayed in Table 2.

Use of PA measures

Of the included 1,382 participants, 1,305 answered questions on PA measures. Of those, 600 (46%) reported that they used any kind of self-report or device-based measures to self-monitor PA. The most common used measure was the simple body worn device, used by 428 (33%), whereof 112 used it regularly. The complex body worn device were used by 182 (14%), whereof 56 used it regularly. One hundred and seven of 1,305 (8%) reported using digital or paper questionnaire and 104 (8%) used digital or paper diaries for self-monitoring of PA. Divided per country, the use of any kind of self-report or device-based measure differed between countries from 34% to 58% (Table 2).

Instructions of PA measures by HPs

Twenty-six of the 428 (6%) users of simple devices received instructions from HPs on how to use the device and of those 182 using the complex device, eight (4%) did. Health professionals instructed 52 (50%) of those 107 using digital/paper questionnaire and 10 out of 104 (10%) of those using diaries.

Perceived importance and confidence in self-monitoring of PA

All the 1,382 answered questions about importance and confidence. Most of the participants, 925 (67%) perceived it was important to measure PA and 558 (40%) reported they did not receive information about PA from any source. Of those receiving information about PA, 388 (28%) received the main source of information from a physiotherapist, 363 (26%) from a rheumatologist, 299 (22%) from internet, 159 (12%) from a general practitioner and 147 (11%)

For peer review only - http://bmjopen.bmj.com/site/about/guidelines.xhtml

from newspapers or magazines. A small number of the study participants received information from friends, 59 (4%), from an occupational therapist 46 (3%) or from a nurse 38 (3%).

The participants were most confident in using and interpreting PA data from simple body worn devices and paper questionnaires. They were least confident in using complex body worn sensors collecting multiple data, as well as using digital diaries (Table 3).

Table 3. Participants confidence/familiarity in using self-reports and devices based physical activity measures (n = 1305)

	**n**	**Median (IQR)**
Simple (0-10)*	1305	3 (8)
Complex sensor (0-10)	1305	1 (6)
Paper QR (0-10)	1305	3 (5)
Paper diary (0-10)	1304	1 (5)
Digital QR (0-10)	1305	1 (5)
Digital diary (0-10)	1305	0 (5)

* possible score range 0-10 0= not confident, 10= very confident
IQR= interquartile range, QR= questionnaire

Fulfilment of physical activity recommendations and its associated factors

A total of 503 of the 1,382 (36%) participants reported they fulfilled the recommendations of ≥ 150 minutes of PA on a moderate intensity per week, whereof 418 (39%) were female.

Univariate logistic regression analyses (Table 4) revealed that those who lived in Ireland (OR = 74.14, P< .001) and Sweden (OR = 1.68, P< .001) were more likely to fulfil the recommendations of PA as compared to those who lived in Denmark, whereas those who lived in Belgium (OR = 0.18, P< .001) were less likely to fulfil the recommendations of PA. The results also showed that participants who were <55 years of age (OR = 1.46, P= .001), working part- or fulltime (OR = 1.61, P< .001), being treated with non-biological disease modifying

For peer review only - http://bmjopen.bmj.com/site/about/guidelines.xhtml

medication (OR = 1.54, P< .001), with no perceived activity limitations in moderate activities (OR = 1.41, P< .001) and who were using a device to measure PA (OR = 1.93, P< .001) were more likely to fulfil the recommendations of PA. In contrast, those of male sex (OR = 0.68, P= .007) and not experiencing pain in the arm (OR = 0.56, P< .001) or leg (OR = 0.65, P< .001) were less likely to fulfil the recommendations of PA.

Table 4. Univariate and multiple logistic regression models for factors associated with fulfilment of recommendations of physical activity.

Variable	*Univariate regression*		*Multiple regression*	
	OR (95% CI)	*P-value*	OR (95% CI)	*P-value*
Country				
Belgium	0.18 (0.14–0.24)	**<.001**	0.21 (0.15–0.29)	**<.001**
Ireland	74.14 (10.22–537.52)	**<.001**	84.89 (11.64–619.23)	**<.001**
Sweden	1.68 (1.13–2.50)	**<.001**	1.68 (1.10–2.56)	**.017**
Denmark	1.00 (reference)		1.00 (reference)	
Age				
<55 years	1.46 (1.74–1.83)	**.001**	1.28 (0.97–1.68)	.076
≥55 years	1.00 (reference)		1.00 (reference)	
Sex				
Male	0.68 (0.51–0.90)	**.007**	0.99 (0.70–1.42)	.971
Female	1.00 (reference)		1.00 (reference)	
Work status				
Working	1.61 (1.28–2.01)	**<.001**	1.41 (0.87–1.50)	.565
Not working	1.00 (reference)		1.00 (reference)	
Education				
Higher education[1]	0.89 (0.81–1.27)	.895		
Basic education[2]	1.00 (reference)			
Bio-DMARDs medication				
Treatment	1.24 (0.97–1.60)	.084		
No treatment	1.00 (reference)			
Non-biological medication				
Treatment	1.54 (1.22–1.94)	**<.001**	0.92 (0.70–1.22)	.565
No treatment	1.00 (reference)		1.00 (reference)	
Activity limitation – moderate activities				
No limitation	1.41 (1.09–1.83)	**<.001**	1.92 (1.38–2.66)	**<.001**
Limitations	1.00 (reference)		1.00 (reference)	
Activity limitation – climbing stairs				

For peer review only - http://bmjopen.bmj.com/site/about/guidelines.xhtml

No limitation	1.00 (0.79–1.27)	.990		
Limitations	1.00 (reference)			
Pain arm				
No pain	0.56 (0.44–0.69)	**<.001**	0.76 (0.57–1.01)	.059
Pain	1.00 (reference)		1.00 (reference)	
Pain hand				
No pain	0.89 (0.72–1.12)	.328		
Pain	1.00 (reference)			
Pain foot				
No pain	1.10 (0.88–1.37)	.407		
Pain	1.00 (reference)			
Pain leg				
No pain	0.65 (0.52–0.81)	**<.001**	1.24 (0.92–1.66)	.153
Pain	1.00 (reference)		1.00 (reference)	
Think it is important to monitor physical activity				
Yes	1.26 (0.99–1.61)	.058		
No	1.00 (reference)			
Use device to measure physical activity				
Yes	1.93 (1.53–2.42)	**<.001**	1.56 (1.20–2.026)	**<.001**
No	1.00 (reference)		1.00 (reference)	
OR: odds ratio, [1]Higher education = university level, [2]Basic education = primary and secondary level				

The multivariable logistic regression model (Table 4) showed that participants who lived in Ireland (OR = 84.89, P< .001) and Sweden (OR = 1.68, P= .017) were more likely to fulfil the recommendations of PA as compared to those who lived in Denmark, whereas those who lived in Belgium (OR = 0.21, P< .001) were less likely to fulfil the recommendations of PA . Furthermore, participants not perceiving activity limitations in moderate activities (OR = 1.92, P< .001), and using a device to measure PA (OR = 1.56, P< .001) remained independent associated factors of fulfilment of recommendations of PA. In this model, 72.5% of the outcome was correctly classified.

DISCUSSION

For peer review only - http://bmjopen.bmj.com/site/about/guidelines.xhtml

To the best of our knowledge this is the first study describing the use of self-report and/or device-based PA measures in individuals with IJD in a real-world situation, across four European countries and its association to fulfilment of PA recommendations.

Overall, close to 50% of the participants used any kind of self-report or device-based measures to self-monitor PA. Furthermore, in our cohort 37% used device-based measures, which is comparable to the general population at the current time (25). This is promising given the motivation for PA which comes with the use of devices and also their advantages of simplicity and accuracy (25, 26). A between country difference of 34% to 58% was observed. It can be considered obvious that younger participants to a larger extent would be those who use wearable trackers. This could be considered supported by the data from Sweden, which has the lowest amount of tracker users, and also is the country with the fewest participants under the age of 55 years. However, Belgium had the highest number of tracker users although the country did not have the youngest group. Hence, other explanations to the between country differences may be sought beyond the variables measured in this study.

Only 112 (8%) of the participants used simple body worn devices and 56 (4%) used complex body worn devices regularly. This is in line with previous interventions aiming to increase PA using wearable trackers in people with rheumatic- and musculoskeletal diseases, showing that adherence to use of trackers over longer periods of time is not maintained (25).

Although a majority (67%) of our sample perceived it was important to measure PA, 40% did not receive information from a HP on why it is important, which is similar to an earlier study in an arthritis population (21). Of those using simple and complex devices only 6% and 4% respectively received instructions on how to use the devices. On the other hand, a substantial

For peer review only - http://bmjopen.bmj.com/site/about/guidelines.xhtml

part, 50%, reported they were instructed how to use questionnaires to measure PA. One explanation to this could be that HPs are used to using traditional questionnaires in clinic and therefore might be more prone to inform their patients about them. Since our participants responded to our survey in 2015-2016, measurement of PA has most likely become more common in clinical practice as well as HPs knowledge about different methods available. One recent study suggest that HPs may be more experienced in using PA devices today (27). The factor most strongly associated with engaging in PA is getting recommendation from a HP and previous research has identified low HP knowledge about PA and exercise as a reason for not guiding patients with IJD in PA (28). Even though HPs are aware of benefits and barriers in relation to PA in IJD, promotion and advice is conflicted by HPs lack of certainty with respect to joint damage and fear of flare-up (29). Moreover, recent studies highlight further educational needs and development for HPs to support PA in this population (19, 30). While PA devices can give the user real-time feedback of daily steps or energy expenditure, patients with IJDs would most likely benefit from also adding HPs support providing them with tailored feed-back to motivate PA (1). In fact, better long-term adherence has been shown in interventions incorporating feedback and behavioural change techniques compared with activity devices alone (31).

The participants' confidence in using self-reports and devices was low. This highlights the importance of HPs communicating with patients on the importance of PA measurement devices to build confidence in their use and their role in changing behaviour to improve PA levels. With HPs increased experience of using device-based measures (27), there is a possibility that communication to patients might increase.

For peer review only - http://bmjopen.bmj.com/site/about/guidelines.xhtml

Both uni- and multivariate regression models showed that participants living in Ireland and Sweden were more likely to fulfil the recommendations of PA as compared to the participants who lived in Denmark, whereas those in Belgium were less likely to fulfil the PA recommendation. This is in line with other studies describing PA levels in these countries (32-34). Furthermore, using a PA device was in addition an independent associated factor of adherence to PA recommendations, also when considering sociodemographic- and disease-related factors, support from HPs, and participants own perception of familiarity and confidence of using PA devices. As this is a cross-sectional study, we cannot determine if the measure itself motivates to PA in this sample, or if the more physically active participants use the measures for self-monitoring of their behaviour. However, using PA devices can be a valuable way to support people with IJDs to improve motivation or PA levels, and it is suggested to be taken into account in the promotion of PA (25, 35, 36). Not perceiving activity limitations was also a factor associated to adherence to PA recommendations in our sample . This corresponds well with a previous review by Veldhuijzen van Zanten et al, where activity limitation is described as a barrier of PA (1). These factors provide information for clinicians to identify which patients need more targeted support to improve their PA levels, an important consideration in managing busy clinical loads.

Limitations

First, the study had a cross-sectional design therefore, conclusions on causal relationships cannot be drawn. This could have been helped by a follow up over time to allow for firmer conclusions about causality.

Second, the questionnaire was based on self-report through social networking, which may have attracted socially desired answers. This is an inherent limitation in most surveys and while it was ensured the data collection was anonymous, self-reports are never free from bias

For peer review only - http://bmjopen.bmj.com/site/about/guidelines.xhtml

(37). Moreover, most of those who answered were those involved in the patient organization, probably with a specific interest in their arthritis, and most likely had a particular interest in the topic. However, as inclusion also took place through outpatient clinics in both Belgium and Sweden, our sample is probably representative of a broader range of characteristics as the patient organizations' members are generally older, thus not working and not on disability leave as they are retired. It should be noted that the overall response rate could not be calculated, as exact numbers of members of the patient organizations were not available or could not be retrieved. Another point which could be regarded as a limitation is that the recruitment procedure through the patient organizations was not similar in each country. In Belgium, Denmark and Ireland invitations to participate were sent via email to members of the patient organizations while in Sweden recruitment took place through the Swedish Rheumatism Association's Facebook page. Furthermore, PA interventions using wearable trackers in people with IJDs have increased the past years (38, 39). This may have promoted increased awareness and use of PA devices since our data was collected. However, we are not aware of other studies which have addressed the same issues as we have. Our data should therefore remain relevant as a starting point for further studies.

As reported earlier in our previous study, investigating rheumatology HPs awareness of PA measures in people with IJDs (30) we cannot neglect the risk of respondents having misinterpreted questions in the survey. Simultaneously with the survey used in the prior study (30) the present survey was designed in English and translated into Swedish, Danish, French and Flemish with results back-translated into English for the reporting. Through the different translations, there is a risk that valuable information was lost. Face validity was investigated in each country before the survey was used, and part of the included questions were other validated questionnaires (23, 40).

For peer review only - http://bmjopen.bmj.com/site/about/guidelines.xhtml

CONCLUSION

Despite these limitations, this study contributes and provides insight into the use of PA measures and devices in a real-world setting by people with IJDs. This is important in order to enhance the collaborative promotion of PA between health professional and patient (31). The study found that there is good use of technology among people with IJDs to measure their own PA signaling the value of such devices in improving overall PA awareness and engagement. Moreover, participants meeting public PA health guidelines were engaged in self-monitoring PA. The study also identified factors associated with PA device use allowing for more targeted management of people with IJDs to improve PA. It also highlights the need for HP engagement with PA advice and understanding of PA devices as an important part of the care of people with IJDs.

Acknowledgement

The authors thank Sara Wiljeminis who assisted in designing the survey and collecting the data.

Contributor ship statement

NB, BN, NK, BAE, and TWS were the original authors who submitted the grant, designed the study, oversaw the data collection and analysis in their countries, prepared the paper and read and commented on all drafts and agreed on the final manuscript. SGMcK, and NMH were research assistants who assisted with designing the survey, collecting the data, and commented on manuscript drafts. DMC and PH undertook the statistical analyses and commented on all manuscript drafts.

Competing interests

None declared

Funding

For peer review only - http://bmjopen.bmj.com/site/about/guidelines.xhtml
For peer review only

The study was funded by the European Leauge Against Rheumatism Health Professional Research Grant 2015.

Data Sharing Statement

No additional data available

Ethics Approval

This study was approved by the Medical Ethical Committee of the University Hospitals Leuven S58849 in Belgium, by the Ethics Committee of the Capital Region of Denmark H-15013386 in Denmark, by the University of Limerick research ethics committee: 2015_09_02_EHS in Ireland and by the Swedish Etikprövningsmyndigheten 2015/1785-31/5 in Sweden.

For peer review only

REFERENCES

1. Veldhuijzen van Zanten JJ, Rouse PC, Hale ED, Ntoumanis N, Metsios GS, Duda JL, et al. Perceived Barriers, Facilitators and Benefits for Regular Physical Activity and Exercise in Patients with Rheumatoid Arthritis: A Review of the Literature. Sports Med. 2015;45(10):1401-12.

2. Collado-Mateo D, Lavín-Pérez AM, Peñacoba C, Del Coso J, Leyton-Román M, Luque-Casado A, et al. Key Factors Associated with Adherence to Physical Exercise in Patients with Chronic Diseases and Older Adults: An Umbrella Review. Int J Environ Res Public Health. 2021;18(4).

3. Caspersen CJ, Powell KE, Christenson GM. Physical activity, exercise, and physical fitness: definitions and distinctions for health-related research. Public health reports (Washington, DC : 1974). 1985;100(2):126-31.

For peer review only - http://bmjopen.bmj.com/site/about/guidelines.xhtml

4. Durcan L, Wilson F, Cunnane G. The effect of exercise on sleep and fatigue in rheumatoid arthritis: a randomized controlled study. The Journal of rheumatology. 2014;41(10):1966-73.

5. Rausch Osthoff AK, Niedermann K, Braun J, Adams J, Brodin N, Dagfinrud H, et al. 2018 EULAR recommendations for physical activity in people with inflammatory arthritis and osteoarthritis. Ann Rheum Dis. 2018;77(9):1251-60.

6. Hootman JM, Macera CA, Ham SA, Helmick CG, Sniezek JE. Physical activity levels among the general US adult population and in adults with and without arthritis. Arthritis Rheum. 2003;49(1):129-35.

7. Tierney M, Fraser A, Kennedy N. Physical activity in rheumatoid arthritis: a systematic review. J Phys Act Health. 2012;9(7):1036-48.

8. Jacquemin C, Servy H, Molto A, Sellam J, Foltz V, Gandjbakhch F, et al. Physical Activity Assessment Using an Activity Tracker in Patients with Rheumatoid Arthritis and Axial Spondyloarthritis: Prospective Observational Study. JMIR mHealth and uHealth. 2018;6(1):e1.

9. Barker J, Smith Byrne K, Doherty A, Foster C, Rahimi K, Ramakrishnan R, et al. Physical activity of UK adults with chronic disease: cross-sectional analysis of accelerometer-measured physical activity in 96 706 UK Biobank participants. International journal of epidemiology. 2019;48(4):1167-74.

10. Haider S, Sedlak M, Kapan A, Grabovac I, Lamprecht T, Erlacher L, et al. Factors Associated with Objectively Measured Physical Activity in Patients with Seropositive Rheumatoid Arthritis. Int J Environ Res Public Health. 2020;17(23).

11. Michie S, Ashford S, Sniehotta FF, Dombrowski SU, Bishop A, French DP. A refined taxonomy of behaviour change techniques to help people change their physical activity and healthy eating behaviours: the CALO-RE taxonomy. Psychology & health. 2011;26(11):1479-98.

For peer review only

12. Page EJ, Massey AS, Prado-Romero PN, Albadawi S. The Use of Self-Monitoring and Technology to Increase Physical Activity: A Review of the Literature. Perspectives on behavior science. 2020;43(3):501-14.

13. Compernolle S, DeSmet A, Poppe L, Crombez G, De Bourdeaudhuij I, Cardon G, et al. Effectiveness of interventions using self-monitoring to reduce sedentary behavior in adults: a systematic review and meta-analysis. The international journal of behavioral nutrition and physical activity. 2019;16(1):63.

14. Patel MS, Asch DA, Volpp KG. Wearable devices as facilitators, not drivers, of health behavior change. Jama. 2015;313(5):459-60.

15. Dowd KP, Szeklicki R, Minetto MA, Murphy MH, Polito A, Ghigo E, et al. A systematic literature review of reviews on techniques for physical activity measurement in adults: a DEDIPAC study. The international journal of behavioral nutrition and physical activity. 2018;15(1):15.

16. Larkin L, Nordgren B, Purtill H, Brand C, Fraser A, Kennedy N. Criterion Validity of the activPAL Activity Monitor for Sedentary and Physical Activity Patterns in People Who Have Rheumatoid Arthritis. Phys Ther. 2016;96(7):1093-101.

17. Arends S, Hofman M, Kamsma YP, van der Veer E, Houtman PM, Kallenberg CG, et al. Daily physical activity in ankylosing spondylitis: validity and reliability of the IPAQ and SQUASH and the relation with clinical assessments. Arthritis Res Ther. 2013;15(4):R99.

18. Smith RD, Dziedzic KS, Quicke JG, Holden MA, McHugh GA, Healey EL. Identification and Evaluation of Self-Report Physical Activity Instruments in Adults With Osteoarthritis: A Systematic Review. Arthritis Care Res (Hoboken). 2019;71(2):237-51.

19. Swärdh E, Opava C, Brodin N. Physical activity in patients with rheumatoid arthritis - an agile lifelong behaviour: a qualitative meta-synthesis. RMD open. 2021;7(2).

For peer review only - http://bmjopen.bmj.com/site/about/guidelines.xhtml

20. Freid LM, Ogdie A, Baker JF. Physical Activity Patterns in People With Inflammatory Arthritis Indicate They Have not Received Recommendation-Based Guidance From Health Care Providers. ACR open rheumatology. 2020;2(10):582-7.

21. Fontaine KR, Bartlett SJ, Heo M. Are health care professionals advising adults with arthritis to become more physically active? Arthritis Rheum. 2005;53(2):279-83.

22. Vandenbroucke JP, von Elm E, Altman DG, Gøtzsche PC, Mulrow CD, Pocock SJ, et al. Strengthening the Reporting of Observational Studies in Epidemiology (STROBE): explanation and elaboration. PLoS medicine. 2007;4(10):e297.

23. Wendel-Vos GC, Schuit AJ, Saris WH, Kromhout D. Reproducibility and relative validity of the short questionnaire to assess health-enhancing physical activity. Journal of clinical epidemiology. 2003;56(12):1163-9.

24. Ainsworth BE, Haskell WL, Leon AS, Jacobs DR, Jr., Montoye HJ, Sallis JF, et al. Compendium of physical activities: classification of energy costs of human physical activities. Medicine and science in sports and exercise. 1993;25(1):71-80.

25. Davergne T, Pallot A, Dechartres A, Fautrel B, Gossec L. Use of Wearable Activity Trackers to Improve Physical Activity Behavior in Patients With Rheumatic and Musculoskeletal Diseases: A Systematic Review and Meta-Analysis. Arthritis Care Res (Hoboken). 2019;71(6):758-67.

26. Sylvia LG, Bernstein EE, Hubbard JL, Keating L, Anderson EJ. Practical guide to measuring physical activity. Journal of the Academy of Nutrition and Dietetics. 2014;114(2):199-208.

27. Leese J, Macdonald GG, Tran BC, Wong R, Backman CL, Townsend AF, et al. Using Physical Activity Trackers in Arthritis Self-Management: A Qualitative Study of Patient and Rehabilitation Professional Perspectives. Arthritis Care Res (Hoboken). 2019;71(2):227-36.

For peer review only - http://bmjopen.bmj.com/site/about/guidelines.xhtml

28. O'Donnell S, Rusu C, Bernatsky S, Hawker G, Canizares M, Mackay C, et al. Exercise/physical activity and weight management efforts in Canadians with self-reported arthritis. Arthritis Care Res (Hoboken). 2013;65(12):2015-23.

29. Halls S, Law RJ, Jones JG, Markland DA, Maddison PJ, Thom JM. Health Professionals' Perceptions of the Effects of Exercise on Joint Health in Rheumatoid Arthritis Patients. Musculoskeletal care. 2017;15(3):196-209.

30. Kennedy NM, McKenna SG, O'Neill A, Appel Esbensen B, Swinnen TW, Nordgren B, et al. A survey across four European countries to determine rheumatology health professionals' awareness of physical activity measures in people with inflammatory joint diseases. BMJ open. 2018;8(5):e020809.

31. Brickwood KJ, Watson G, O'Brien J, Williams AD. Consumer-Based Wearable Activity Trackers Increase Physical Activity Participation: Systematic Review and Meta-Analysis. JMIR mHealth and uHealth. 2019;7(4):e11819.

32. McKenna S, Tierney M, O'Neill A, Fraser A, Kennedy N. Sleep and physical activity: a cross-sectional objective profile of people with rheumatoid arthritis. Rheumatol Int. 2018;38(5):845-53.

33. Bremander A, Malm K, Andersson ML. Physical activity in established rheumatoid arthritis and variables associated with maintenance of physical activity over a seven-year period - a longitudinal observational study. BMC rheumatology. 2020;4:53.

34. Rasmussen JO, Primdahl J, Fick W, Bremander A. Physical activity in people with axial spondyloarthritis and the impact of overall attitudes, barriers, and facilitators: A cross-sectional study. Musculoskeletal care. 2020;18(4):510-8.

35. Li C, Chen X, Bi X. Wearable activity trackers for promoting physical activity: A systematic meta-analytic review. International journal of medical informatics. 2021;152:104487.

For peer review only

For peer review only - http://bmjopen.bmj.com/site/about/guidelines.xhtml

36. Ashur C, Cascino TM, Lewis C, Townsend W, Sen A, Pekmezi D, et al. Do Wearable Activity Trackers Increase Physical Activity Among Cardiac Rehabilitation Participants? A SYSTEMATIC REVIEW AND META-ANALYSIS. Journal of cardiopulmonary rehabilitation and prevention. 2021;41(4):249-56.

37. van de Mortel TF. Faking it: social desirability response bias in self-report research. Australian Journal of Advanced Nursing. 2008;25(4):40-8.

38. Davergne T, Rakotozafiarison A, Servy H, Gossec L. Wearable Activity Trackers in the Management of Rheumatic Diseases: Where Are We in 2020? Sensors (Basel, Switzerland). 2020;20(17).

39. Pellegrini CA, Powell SM, Mook N, DeVivo K, Ehrlich-Jones L. Use of Physical Activity Monitors in Rheumatic Populations. Current rheumatology reports. 2018;20(12):73.

40. Linton SJ, Halldén K. Can we screen for problematic back pain? A screening questionnaire for predicting outcome in acute and subacute back pain. The Clinical journal of pain. 1998;14(3):209-15.

For peer review only

For peer review only - http://bmjopen.bmj.com/site/about/guidelines.xhtml

Supplementary file – Survey

A survey of people with inflammatory joint diseases use of physical activity measures

Demographics

* 1. Gender

- Male
- Female

* 2. What is your age?

- 18 to 24
- 25 to 34
- 35 to 44
- 45 to 54
- 55 to 64
- 65 to 74
- 75 or older

* 3. What type of arthritis have you been diagnosed with?

- Rheumatoid Arthritis (RA)
- Ankylosing Spondylitis (AS)
- Psoriatic Arthritis (PsA)

Other (please specify)

* 4. What year did you first experience your symptoms?

For peer review only

* 5. What body region do you have pain in?

- [ ] Arm
- [ ] Hand
- [ ] Foot
- [ ] Leg
- [ ] Spine
- [ ] Neck
- [ ] Head
- [ ] Other

Other (please specify)

6. Does your health limit you in the following activities?

	Yes, limited a lot	Yes, limited a little	No, not limited at all
Moderate activities e.g. moving a table, vacuum cleaning, bowling, golf etc.	☐	☐	☐
Climbing several flights of stairs	☐	☐	☐

For peer review only

* 7. What type of medication are you on?

- I do not take medications
- Biological DMARDS
- Non-biological DMARDS
- I do not know

Other (please specify)

* 8. What is your working situation?

- Full-time employment
- Part-time employment
- Unemployed
- Retired (due to age)
- Disability
- Housewife/Househusband

Other (please specify)

* 9. What is your highest level of education?

- Primary level
- Secondary level
- Third level

Other (please specify)

For peer review only

Views on measuring Physical Activity

* 10. Do you think it is important to measure physical activity?

- Yes
- No

11. Why do you/don't you think it is important to measure physical activity?

12. Where do you find your information on measuring physical activity?

- [ ] I don't
- [ ] Physiotherapist
- [ ] Rheumatologist
- [ ] Occupational Therapist
- [ ] Nurse
- [ ] Friends
- [ ] GP/Family Doctor
- [ ] Internet
- [ ] Magazine/Newspaper

Other (please specify)

Physical Activity Measurement - Objective measurement

Physical Activity is important for people with IJD's. While the measurement and monitoring of same can be a challenge, it has a number of essential uses. The following questions are concerned with the more popular ways in which Physical Activity is monitored and your answers will guide the evidence base and practice of health promotion.

13. How familiar are you with the following devices/ways in monitoring physical activity? (0 is not familiar and 10 is very familiar)

For peer review only

0 1 2 3 4 5 6 7 8 9 10

PEDOMETER

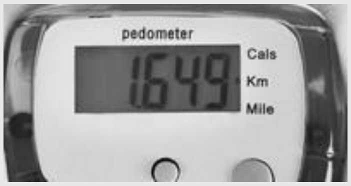

ACCELEROMETER

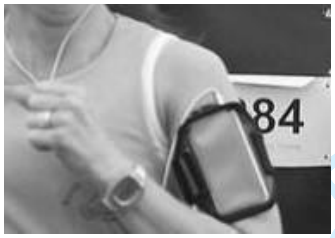

INTERNET

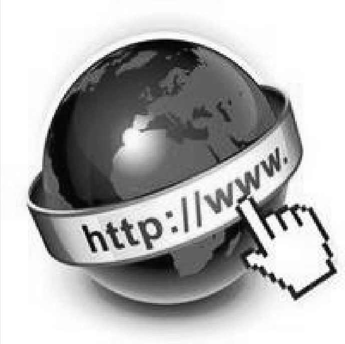

SMARTPHONE APP

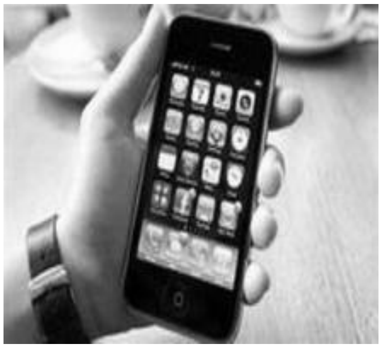

	0	1	2	3	4	5	6	7	8	9	10
QUESTIONNAIRE	○	○	○	○	○	○	○	○	○	○	○
DIARY	○	○	○	○	○	○	○	○	○	○	○

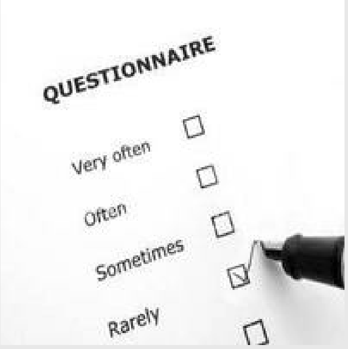

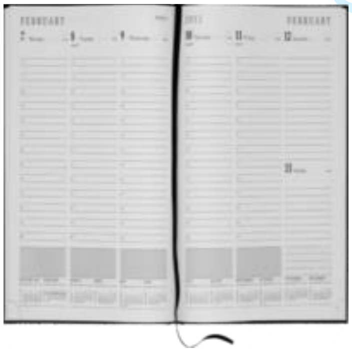

14. The following questions are in relation to PEDOMETERS

How familiar are you in using a Pedometer to measure Physical Activity?

- ○ Very familiar
- ○ Somewhat familiar
- ○ Vaguely familiar
- ○ Not familiar

15. Do you own a Pedometer?

- ○ Yes
- ○ No

For peer review only

16. If Yes, where did you get the Pedometer?

- ◯ I bought it
- ◯ A gift
- ◯ Provided by my employer/work place
- ◯ From a study
- ◯ From a Health Professional

Other (please specify)

17. If Yes, how frequently do you wear a Pedometer?

- ◯ Every Day
- ◯ Most Days
- ◯ Rarely wear it
- ◯ Never wear it

18. Have you been instructed in how to use a Pedometer by a Health Professional within Rheumatology?

- ◯ Yes
- ◯ No

19. If Yes, who has instructed you in using it?

- ◯ Nurse
- ◯ Rheumatologist
- ◯ Physiotherapist
- ◯ Occupational Therapist

Other (please specify)

For peer review only

20. How familiar are you in reading the results from a Pedometer?

- Very familiar
- Somewhat familiar
- Vaguely familiar
- Not familiar

21. The following questions are in relation to ACCELEROMETERS

How familiar are you in using an Accelerometer for measuring Physical Activity?

- Very familiar
- Somewhat familiar
- Vaguely familiar
- Not familiar

22. Do you own an Accelerometer?

- Yes
- No

23. If Yes, where did you get the Accelerometer?

- I bought it
- A gift
- Provided by my employer/work place
- From a study
- From a Health Professional

Other (please specify)

For peer review only

24. If Yes, how frequently do you wear an Accelerometer?

- Every Day
- Most Days
- Rarely wear it
- Never wear it

25. Have you been instructed in how to use an Accelerometer by a Health Professional within Rheumatology?

- Yes
- No

26. If Yes, who has instructed you in using it?

- Nurse
- Rheumatologist
- Physiotherapist
- Occupational Therapist

Other (please specify)

27. How familiar are you in reading the results from an Accelerometer?

- Very familiar
- Somewhat familiar
- Vaguely familiar
- Not familiar

28. The following questions are in relation to the INTERNET

How familiar are you in using the INTERNET for measuring Physical Activity?

- Very familiar
- Somewhat familiar
- Vaguely familiar
- Not familiar

For peer review only

29. Do you use the INTERNET for measuring Physical Activity?

- Yes
- No

30. If Yes, have any of the following recommended you to use it?

- Nurse
- Rheumatologist
- Physiotherapist
- Occupational Therapist

Other (please specify)

31. If Yes, how frequently do you use the INTERNET to measure your Physical Activity?

- Every Day
- Most Days
- Rarely wear it
- Never wear it

32. Have you been instructed in how to use the INTERENET to measure your Physical Activity by a Health Professional within Rheumatology?

- Yes
- No

33. If Yes, who has instructed you in using it?

- Nurse
- Rheumatologist
- Physiotherapist
- Occupational Therapist

Other (please specify)

For peer review only

34. How familiar are you in reading the results of your Physical Activity from the INTERNET?

- ◯ Very familiar
- ◯ Somewhat familiar
- ◯ Vaguely familiar
- ◯ Not familiar

35. The following questions are in relation to SMARTPHONE APPS

How familiar are you in using a Smartphone App for measuring Physical Activity?

- ◯ Very familiar
- ◯ Somewhat familiar
- ◯ Vaguely familiar
- ◯ Not familiar

36. Do you have a Smartphone App for Physical Activity?

- ◯ Yes
- ◯ No

For peer review only

37. If Yes, where did you get your Smartphone App?

- I downloaded it - Free
- I downloaded it - Paid
- A gift
- Provided by my employer/work place
- From a study
- From a Health Professional

Other (please specify)

38. If Yes, how frequently do you use a Smartphone App?

- Every Day
- Most Days
- Rarely wear it
- Never wear it

39. Have you been instructed in how to use a Smartphone App by a Health Professional within Rheumatology?

- Yes
- No

40. If Yes, who has instructed you in using it?

- Nurse
- Rheumatologist
- Physiotherapist
- Occupational Therapist

Other (please specify)

For peer review only

41. How familiar are you in reading the results from a Smartphone App?

- Very familiar
- Somewhat familiar
- Vaguely familiar
- Not familiar

42. The following questions are in relation to QUESTIONNAIRES

How familiar are you in using a Questionnaire or Diary for measuring Physical Activity?

- Very familiar
- Somewhat familiar
- Vaguely familiar
- Not familiar

43. Do you use a Questionnaire for measuring Physical Activity?

- Yes
- No

44. If Yes, have any of the following recommended you to use it?

- Nurse
- Rheumatologist
- Physiotherapist
- Occupational Therapist

Other (please specify)

For peer review only

45. If Yes, how frequently do you use a Questionnaire to measure your Physical Activity?

- ◯ Every Day
- ◯ Most Days
- ◯ Rarely wear it
- ◯ Never wear it

46. How familiar are you in reading the results of your Physical Activity from a Questionnaire?

- ◯ Very familiar
- ◯ Somewhat familiar
- ◯ Vaguely familiar
- ◯ Not familiar

47. The following questions are in relation to DIARIES

How familiar are you in using a Diary for measuring Physical Activity?

- ◯ Very familiar
- ◯ Somewhat familiar
- ◯ Vaguely familiar
- ◯ Not familiar

48. Do you use a Diary for measuring Physical Activity?

- ◯ Yes
- ◯ No

49. If Yes, have any of the following recommended you to use it?

- ◯ Nurse
- ◯ Rheumatologist
- ◯ Physiotherapist
- ◯ Occupational Therapist

Other (please specify)

For peer review only

50. If Yes, how frequently do you use a Diary to measure your Physical Activity?

- Every Day
- Most Days
- Rarely wear it
- Never wear it

51. How familiar are you in reading the results of your Physical Activity from a Diary?

- Very familiar
- Somewhat familiar
- Vaguely familiar
- Not familiar

Barriers and Preferences

52. Do you think there are any barriers to people with arthritis measuring their own physical activity levels?

- Yes
- No

53. If Yes, please give an example

54. In your opinion, what is the best way to measure Physical Activity for people with arthritis?

* Pedometer
* Accelerometer
* Internet
* Smartphone app
* Questionnaire
* Diary

For peer review only

Current Physical Activity Levels

* 55. Think about an average week in the past few months and please answer the following regarding yourown Physical activites

	How many days per week?	Average time in minutes per day?	Intensity of activity
Commuting Activities i.e. Walk, Cycle etc.			
Work Activities i.e. Walking, Standing, Lifting etc.			
Leisure Time Activities i.e. Walking, Cycling, Gardening etc.			
Sport Activities i.e. Tennis, Football, Rugby etc.			
Household Activities i.e. Cleaning, Washing etc.			

For peer review only

STROBE 2007 (v4) Statement—Checklist of items that should be included in reports of *cross-sectional studies*

Section/Topic	Item #	Recommendation	Reported on page #
**Title and abstract**	1	(*a*) Indicate the study's design with a commonly used term in the title or the abstract	1-3
		(*b*) Provide in the abstract an informative and balanced summary of what was done and what was found	2
**Introduction**			
Background/rationale	2	Explain the scientific background and rationale for the investigation being reported	3-4
Objectives	3	State specific objectives, including any prespecified hypotheses	4
**Methods**			
Study design	4	Present key elements of study design early in the paper	5
Setting	5	Describe the setting, locations, and relevant dates, including periods of recruitment, exposure, follow-up, and data collection	5-7
Participants	6	(*a*) Give the eligibility criteria, and the sources and methods of selection of participants	5
Variables	7	Clearly define all outcomes, exposures, predictors, potential confounders, and effect modifiers. Give diagnostic criteria, if applicable	5-6
Data sources/ measurement	8*	For each variable of interest, give sources of data and details of methods of assessment (measurement). Describe comparability of assessment methods if there is more than one group	5-8
Bias	9	Describe any efforts to address potential sources of bias	Supplementary file
Study size	10	Explain how the study size was arrived at	-
Quantitative variables	11	Explain how quantitative variables were handled in the analyses. If applicable, describe which groupings were chosen and why	8
Statistical methods	12	(*a*) Describe all statistical methods, including those used to control for confounding	8
		(*b*) Describe any methods used to examine subgroups and interactions	8
		(*c*) Explain how missing data were addressed	-
		(*d*) If applicable, describe analytical methods taking account of sampling strategy	-
		(*e*) Describe any sensitivity analyses	-
**Results**			

For peer review only

Participants	13*	(a) Report numbers of individuals at each stage of study—eg numbers potentially eligible, examined for eligibility, confirmed eligible, included in the study, completing follow-up, and analysed	8-9
		(b) Give reasons for non-participation at each stage	-
		(c) Consider use of a flow diagram	-
Descriptive data	14*	(a) Give characteristics of study participants (eg demographic, clinical, social) and information on exposures and potential confounders	Table 2
		(b) Indicate number of participants with missing data for each variable of interest	8-9, Table 2
Outcome data	15*	Report numbers of outcome events or summary measures	
Main results	16	(*a*) Give unadjusted estimates and, if applicable, confounder-adjusted estimates and their precision (eg, 95% confidence interval). Make clear which confounders were adjusted for and why they were included	Table 5
		(*b*) Report category boundaries when continuous variables were categorized	Table 1
		(*c*) If relevant, consider translating estimates of relative risk into absolute risk for a meaningful time period	Not applicable
Other analyses	17	Report other analyses done—eg analyses of subgroups and interactions, and sensitivity analyses	-
**Discussion**			
Key results	18	Summarise key results with reference to study objectives	10-11
Limitations	19	Discuss limitations of the study, taking into account sources of potential bias or imprecision. Discuss both direction and magnitude of any potential bias	13
Interpretation	20	Give a cautious overall interpretation of results considering objectives, limitations, multiplicity of analyses, results from similar studies, and other relevant evidence	10-12 + 14
Generalisability	21	Discuss the generalisability (external validity) of the study results	12, 13
**Other information**			
Funding	22	Give the source of funding and the role of the funders for the present study and, if applicable, for the original study on which the present article is based	15

*Give information separately for cases and controls in case-control studies and, if applicable, for exposed and unexposed groups in cohort and cross-sectional studies.

Note: An Explanation and Elaboration article discusses each checklist item and gives methodological background and published examples of transparent reporting. The STROBE checklist is best used in conjunction with this article (freely available on the Web sites of PLoS Medicine at http://www.plosmedicine.org/, Annals of Internal Medicine at http://www.annals.org/, and Epidemiology at http://www.epidem.com/). Information on the STROBE Initiative is available at www.strobe-statement.org.

For peer review only

BMJ Open

Self-report and device-based physical activity measures and adherence to physical activity recommendations. A cross-sectional survey among people with inflammatory joint disease in four European countries.

Journal:	*BMJ Open*
Manuscript ID	bmjopen-2022-064278.R2
Article Type:	Original research
Date Submitted by the Author:	24-Jan-2023
Complete List of Authors:	Brodin, N; Karolinska Institutet, Department of Neurobiology Care Sciences and Society, Division of Physiotherapy; Danderyds Sjukhus AB, Division of Physiotherapy, Orthopaedic Clinic<br>Conradsson, David; Karolinska Institutet Department of Neurobiology Care Sciences and Society, Division of Physiotherapy; Karolinska University Hospital, Medical unit Occupational therapy & Physiotherapy, Theme Women's Health and Allied Health Professional<br>Swinnen, Thijs; KU Leuven, Department of Rehabilitation Sciences; KU Leuven University Hospitals Leuven, Division of Rheumatology<br>Esbensen, Bente; Rigshospitalet; University of Copenhagen, Department of Clinical Medicine<br>Kennedy, Norelee; University of Limerick, Discipline of Physiotherapy, School of Allied Health; University of Limerick, Health Research Institute,<br>Hammer, Nanna Maria ; Rigshospitalet, Copenhagen Center for Arthritis Research (Copecare)<br>McKenna, Sean; University of Limerick, Discipline of Physiotherapy, School of Allied Health; University of Limerick Hospitals Group, Health Service Executive<br>Henriksson, Peter ; Karolinska Institutet, Department of clinical sciences, Danderyds hospital<br>Nordgren, Birgitta; Karolinska Institutet Department of Neurobiology Care Sciences and Society, Division of Physiotherapy; Karolinska University Hospital, Medical unit Occupational therapy & Physiotherapy, Theme Women's Health and Allied Health Professional
**Primary Subject Heading**:	Rheumatology
Secondary Subject Heading:	Evidence based practice
Keywords:	Musculoskeletal disorders < ORTHOPAEDIC & TRAUMA SURGERY, Rheumatology < INTERNAL MEDICINE, Health informatics < BIOTECHNOLOGY & BIOINFORMATICS

SCHOLARONE™
Manuscripts

I, the Submitting Author has the right to grant and does grant on behalf of all authors of the Work (as defined in the below author licence), an exclusive licence and/or a non-exclusive licence for contributions from authors who are: i) UK Crown employees; ii) where BMJ has agreed a CC-BY licence shall apply, and/or iii) in accordance with the terms applicable for US Federal Government officers or employees acting as part of their official duties; on a worldwide, perpetual, irrevocable, royalty-free basis to BMJ Publishing Group Ltd ("BMJ") its licensees and where the relevant Journal is co-owned by BMJ to the co-owners of the Journal, to publish the Work in this journal and any other BMJ products and to exploit all rights, as set out in our licence.

The Submitting Author accepts and understands that any supply made under these terms is made by BMJ to the Submitting Author unless you are acting as an employee on behalf of your employer or a postgraduate student of an affiliated institution which is paying any applicable article publishing charge ("APC") for Open Access articles. Where the Submitting Author wishes to make the Work available on an Open Access basis (and intends to pay the relevant APC), the terms of reuse of such Open Access shall be governed by a Creative Commons licence – details of these licences and which Creative Commons licence will apply to this Work are set out in our licence referred to above.

Other than as permitted in any relevant BMJ Author's Self Archiving Policies, I confirm this Work has not been accepted for publication elsewhere, is not being considered for publication elsewhere and does not duplicate material already published. I confirm all authors consent to publication of this Work and authorise the granting of this licence.

For peer review only

Self-report and device-based physical activity measures and adherence to physical activity recommendations. A cross-sectional survey among people with inflammatory joint disease in four European countries.

Brodin N[1,2] Moulaee Conradsson D [1,3], Swinnen TWS [4,5], Esbensen B.A [6,7], Kennedy N [8,9], Hammer NM[6], McKenna S[8,10], Henriksson P[11], Nordgren B[1,3]

Authors affiliation

1 Division of Physiotherapy, Department of Neurobiology, Care Sciences and Society, Karolinska Institutet, Stockholm, Sweden

2 Division of Physiotherapy, Orthopaedic Clinic, Danderyds Sjukhus Corp.,, Stockholm, Sweden

3 Medical unit Occupational therapy & Physiotherapy, Theme Women's Health and Allied Health Professional, Karolinska University Hospital, Stockholm, Sweden

4 Department of Rehabilitation Sciences, Research Group for Musculoskeletal Rehabilitation, KU Leuven, Leuven, Belgium

5 Division of Rheumatology, UZ Leuven, Leuven, Belgium

6 Copenhagen Center for Arthritis Research, Center for Rheumatology and Spine Diseases, Centre of Head and Orthopaedics, Rigshospitalet, Glostrup, Denmark

7 Department of Clinical Medicine, University of Copenhagen, Copenhagen, Denmark

8 Discipline of Physiotherapy, School of Allied Health, University of Limerick, Limerick, Ireland

9 Health Research Institute, University of Limerick, Limerick, Ireland

10 Health Service Executive, University Hospitals Limerick Group, Limerick, Ireland

11 Department of Clinical Sciences Danderyd Hospital, Karolinska Institutet, Stockholm, Sweden

Corresponding Author

Dr Birgitta Nordgren

Division of Physiotherapy, Department of Neurobiology, Care Sciences and Society, Karolinska Institutet, Stockholm, Sweden

For peer review only - http://bmjopen.bmj.com/site/about/guidelines.xhtml

Medical unit Occupational therapy & Physiotherapy, Theme Women's Health and Allied Health Professional, Karolinska University Hospital, Stockholm, Sweden

birgitta.nordgren@ki.se

For peer review only

ABSTRACT

Objectives: Self-monitoring of physical activity (PA) has the potential to contribute to successful behaviour change in PA interventions in different populations, including people with inflammatory joint diseases (IJDs). The objectives of this study were to describe the use and knowledge of self-report- and device-based PA measures in people with IJDs in four European countries, and to explore if the use of such devices, socio-demographic or disease-related variables were associated with adherence to the recommendations of at least 150 minutes of moderate to vigorous PA per week.

Setting: Cross-sectional survey, performed in 2015-2016.

Participants: People with IJDs in Belgium, Denmark, Ireland and Sweden.

Primary and secondary outcome measures: Use of self-report and device-based PA measures, receipt of instructions how to use PA measures, confidence in using them, adherence to PA recommendations and associated factors for adherence to PA recommendations.

Results: Of the 1,305 respondents answering questions on PA measures, 600 (46%) reported use of any kind of self-report or device-based measures to self-monitor PA. Between country differences of 34% to 58% was observed. Six and four percent received instructions from health professionals on how to use simple and complex devices, respectively. Independent associated factors of fulfilment of recommendations of PA were living in Ireland (OR = 84.89, $P< .001$) and Sweden (OR = 1.68, $P= .017$) compared to living in Denmark, not perceiving activity limitations in moderate activities (OR = 1.92, $P< .001$) and using a device to measure PA (OR = 1.56, $P< .001$). Those living in Belgium (OR = 0.21, $P< .001$) were less likely to fulfil recommendations of PA.

Conclusions: Almost half of the participants with IJDs used self- or deviced based PA measures, although few used wearable devices regularly. The results indicate that participants meeting public PA health guidelines were engaged in self-monitoring of PA.

For peer review only - http://bmjopen.bmj.com/site/about/guidelines.xhtml

Strengths and limitations of the study

- First study describing the use of self-report and/or device-based PA measures in people with IJD in a real-world setting across four European countries
- Study participants had low confidence in using device-based PA measures
- Study participants might have been particularly interested in PA prompting their participation in the study, limiting external validity
- Due to the cross-sectional design, conclusions on causal relationships cannot be drawn

INTRODUCTION

Regular physical activity (PA) is associated with lower risk of cardiovascular disease, diabetes and several cancers. Healthy behaviours such as achieving sufficient PA is a challenge for most people but may be even more challenging for people with inflammatory joint diseases (IJDs), as IJDs are characterized by functional limitations, pain and fatigue (1, 2).

PA defined as "any bodily movement produced by skeletal muscles that result in energy expenditure" (3) is safe and can improve disease activity, pain, fatigue, quality of life, and sleep in people with IJDs (4, 5). Despite these beneficial health outcomes, people with IJDs have lower PA levels than their healthier counterparts and many do not meet the required PA recommendations (6-10).

Self-monitoring of PA has the potential to contribute to successful behaviour change in PA interventions in different populations, including people with IJDs (11-14). Physical activity can be monitored in different ways, although measurement properties (i.e., validity, reliability and

For peer review only - http://bmjopen.bmj.com/site/about/guidelines.xhtml

responsiveness to change) vary considerably across techniques. Common methods for monitoring PA range from simple questionnaires and pedometers to more complex devices such as accelerometers (15). Self-reported measures such as diaries and questionnaires as well as device-based PA measures can raise awareness and motivate patients to initiate and maintain PA (12). In particular, device-based wearable trackers can give the user valid data on, for example, daily steps or PA intensity levels (16). Given their superior feasibility, adequate reliability and sufficient construct validity in group analyses, self-reported PA questionnaires remain useful to relate PA behaviours to clinical outcome in larger arthritis research studies (17, 18). Moreover, health professionals (HP) play an important role in supporting PA behavior in people with IJDs and could contribute to better health outcomes through promoting PA monitoring in clinical practice (19-21). Better knowledge and understanding of people with IJDs use and awareness of PA measures and its association to PA levels is needed to guide future practice of health promotion in this population. The use of PA measures in different European countries and to what extent people with IJDs perceive it to be important in measuring PA behaviour needs to be further explored.

For peer review only

The main objective of this study was therefore to describe the use, knowledge and sources of information of self-report- and device-based PA measures in people with IJD in four European countries. A further objective was to explore if the use of such devices, socio-demographic factors or disease-related variables were associated with adherence to the recommendations of at least 150 minutes of moderate to vigorous PA per week.

METHODS

Design

Cross-sectional design using questionnaire was chosen as it allowed for the collection of data

For peer review only - http://bmjopen.bmj.com/site/about/guidelines.xhtml

from a wider range of participants. The STROBE reporting guidelines for observational studies were used to guide the reporting of this study (22).

Patient and Public Involvement

Patients representing different IJDs in the four countries were involved in the design of the questionnaire. This was organized following the same structure in all countries. The patients were invited by each country representative to give their views and input on the questionnaire. They were asked to comment on the content of the questionnaire and identify any missing or problematic questions/constructs. All input was considered in the final questionnaire.

Sample

People with IJDs in four European countries, i.e., Belgium, Denmark, Ireland and Sweden were invited to participate in the study. In Belgium, people were identified through an outpatient university clinic and through patient organisations. In Denmark and Ireland, people were identified through the country's patient organisation membership. In Sweden, recruitment was performed through a Facebook group hosted by the patient organisation and from an outpatient rheumatology clinic. The chairperson for each country's patient organisation was contacted, requesting permission for their members to participate in the study. Following the granted permission, the chairpersons in each country received information via email, containing study information, survey link and researcher details and they provided the members with the same. In Sweden, participants recruited at the rheumatology clinic received the same information mentioned above as well as the paper questionnaire distributed through the patient organisation. Consent to participate was provided by responding to the survey anonymously. Detailed information on the study was provided on the first page of the survey. Separate ethical approvals

For peer review only - http://bmjopen.bmj.com/site/about/guidelines.xhtml

were approved by each participating country's relevant research ethics committees (Belgium: S58849, Denmark: H-15013386, Ireland: 2015_09_02_EHS, Sweden: 2015/1785-31/5).

Questionnaire

The study steering group developed a questionnaire divided into four domains: 1) sociodemographic; 2) disease-related factors, 3) PA self-monitoring measures; questionnaire and diary (paper or digital), simple body worn PA devices, (e.g., simple wearable activity trackers), or complex body worn devices (e.g., accelerometer, and wearable activity trackers with wireless link to smartphone app or website) and 4) questions about receiving instructions how to use PA measures, the importance of measuring PA, confidence in using PA measures; and if having been recommended by an HP to self-monitor PA using any self-report or device-derived measure (online supplementary file).

The questionnaire was developed in English and translated into each country's language. To ascertain face validity of each country's version of the questionnaire, discussions were organised with health professionals' i.e., Physiotherapist, Occupational Therapist and a Clinical Nurse Specialist. In addition, interviews with four people diagnosed with different IJD (Rheumatoid Arthritis (RA) n=2, Ankylosing Spondylitis (AS) n=1 and Psoriatic Arthritis (PSA) n=1)) to cover different disease spectra were also organised country-wise. These discussions took place to explore whether the constructs surveyed within each questionnaire reflected the aims under study (i.e., to identify missing or problematic questions/constructs) and if the questions were well understood. We therefore applied a purposive sampling method to reflect different diagnoses of arthritis, age, and gender and subsequently the questionnaires were adapted to improve their readability, validity and specificity. The final questionnaires were piloted with people with IJD and experts in this field to ensure content validity. Thus, verifying

For peer review only - http://bmjopen.bmj.com/site/about/guidelines.xhtml

whether questions were readable, relevant and representative of the study's aims, in addition to minimise the risk of missing data.

PA levels were measured using the The Short Questionnaire to Assess Health Enhancing Physical Activity (SQUASH) questionnaire (23). The SQUASH allows a comparison of national and international PA recommendations and includes questions on commuting activities (walking, cycling), PA at work or school, household activities and leisure time activities (walking, gardening, cycling, sports). The participants were asked to refer to a normal week during the last month; how many days a week they engaged in each of the activities, the average time per day spent on each activity (hours and minutes) and the effort of each activity (low, moderate, high). Activities were assigned a Metabolic Equivalent Task value (MET) according to Ainsworth's' compendium of physical activities (24). Based on reported effort of the activities in the questionnaire, activities were assigned an intensity score between 1-9, were ≥ 3 assumed to represent health-enhancing PA. Study participants, who reported 150 min or more of moderate or more intensive activities per week, were classified as reaching health-enhancing PA recommendations. This was calculated by summing up the number of days per week for activities on a moderate or higher intensity level where the total duration was 30 minutes or more. A minimum of five days resulted in patients being categorized as reaching the health-enhancing PA recommendations (5). The SQUASH has previously demonstrated good test–retest reliability and modest construct validity in the IJD population (17).

Data Collection

The survey was conducted online during 2015-2016 with one reminder sent. For participants in Belgium, Denmark, and Ireland the survey was distributed through SurveyMonkey or

For peer review only - http://bmjopen.bmj.com/site/about/guidelines.xhtml

SurveyXact. In Sweden the survey was conducted online through Artologik Survey&Report as well as through paper questionnaire distributed at an outpatient rheumatology clinic.

Analysis

Statistical analyses were carried out using IBM SPSS, version 27.0 (SPSS Inc., Chicago, Illinois, USA).

For the main objective, the results were presented descriptively using proportions and percentage or median and inter quartile range (IQR) for categorical variables and means and standard deviations (SD) for continuous variables.

For further objectives, Table 1 display variables and criteria used for categorization of independent and dependent variables for logistic regressions.

Table 1. Variables and criteria used for categorization of the independent variables and the dependent variable.

Variables and instruments	Criteria for categorization
Independent variables	
Country	Belgium/ Denmark/Ireland/Sweden//
Age	≥55 years/<55 years
Sex	Female/male
Work status	Working/not working
Education[1]	Higher/basic
Bio-DMARDs medication	Treatment/No treatment
Non-biol medication	Treatment/No treatment
Activity limitation - moderate activities	No limitation/Limitations
Activity limitation -stairs	No limitation/Limitations
Pain arm	No pain/pain
Pain hand	No pain/pain

For peer review only - http://bmjopen.bmj.com/site/about/guidelines.xhtml

Pain foot	No pain/pain
Pain leg	No pain/pain
Think it is important to monitor physical activity	Yes/No
Use device measuring physical activity	Yes/No
Dependent variable	
≥150 minutes a week of moderate –high intensity physical activity	Yes/No

[1]Higher education = university level. Basic education = primary and secondary level

Firstly, potential associated factors for fulfilment of PA recommendation were entered one at a time in unadjusted univariate logistic regression models. At this point, the cut-off to include factors in the multivariate analyses was set to an alpha level P≤ 0.1. Prior to multivariable analyses, measures of association, i.e., Chi Square tests, were computed to assess for collinearity among potential associated factors. Associated factors were subsequently entered into a multivariable logistic regression model with an alpha level set at P≤ 0.05 for the identification of independent associated factors. Results for logistic regressions are expressed as odds ratios (OR) and 95% confidence intervals (95% CI).

RESULTS

Study participants

A total of 1,382 participants with IJD in Belgium, Denmark, Ireland and Sweden answered the survey mainly online. A majority of the participants were diagnosed with RA (n = 554, 40%), 331 (24%) with AS, 296 (21%) with PSA and 201 (15%) with other diagnoses (Table 2).

Table 2 Demographic and disease-related factors and fulfilment of physical activity recommendations of participants

For peer review only - http://bmjopen.bmj.com/site/about/guidelines.xhtml

Variables	**Belgium** (n=591)	**Denmark** (n=579)	**Ireland** (n=90)	**Sweden** (n=122)	**Total** (n=1382)
Diagnosis n (%)					
RA	217 (37)	237 (41)	56 (62)	44 (36)	554 (40)
AS	221 (37)	77 (13)	13 (14)	20 (16)	331(24)
PSA	101 (17)	162 (28)	15 (17)	18 (15)	296 (21)
Other[1]	52 (9)	103 (18)	6 (7)	40 (33)	201 (15)
Female sex n (%)	409 (69)	509 (88)	69 (77)	108 (86)	1095 (79)
Age, < 55years (%)	263 (45)	347 (60)	48 (53)	48 (39)	706 (51)
Years symptom, mean (SD)	17.9 (12.4)	13.7 (10.8)	10.1 (7.9)	15.2 (12.2)	15.4 (11.8)
Bio-DMARDs medication (%)	150 (25)	136 (24)	39 (43)	48 (40)	373 (27)
Non-biological medication n (%)	105 (18)	306 (53)	26 (29)	39 (32)	476 (34)
Basic education[2] n (%)	289 (49)	259 (45)	37 (41)	60 (49)	645 (48)
Working full/part time n (%)	203 (34)	234 (40)	44 (49)	70 (57)	551 (40)
Pain					
Hand n (%)	285 (48)	371 (64)	40 (44)	81 (66)	777 (56)
Arm n (%)	149 (25)	327 (57)	50 (56)	79 (65)	605 (44)
Foot n (%)	248 (42)	306 (53)	31 (34)	69 (57)	654 (47)
Leg n (%)	135 (23)	335 (58)	43 (48)	95 (79)	608 (44)
Back n (%)	323 (55)	307 (53)	18 (20)	66 (54)	714 (52)
Neck n (%)	271 (46)	219 (38)	15 (17)	49 (40)	554 (40)
Head n (%)	80 (14)	55 (9)	2 (2)	12 (9)	149 (11)
Activity limitation					
Limited a lot moderate activities n (%)	113 (19)	156 (27)	21 (23)	17 (14)	307 (22)
Limited a lot climbing stairs n (%)	60 (10)	147 (25)	26 (29)	20 (16)	253 (18)
Own or use PA device n (%)	340 (58)	274 (47)	49 (54)	42 (34)	600 (43)
Fulfilment of PA recommendations n (%)	84 (14)	277 (48)	68 (76)	74 (61)	503 (36)

RA: Rheumatoid arthritis, AS: Ankylosing spondylitis, PSA: Psoriatic arthritis, SD: Standard deviation, PA: Physical activity,
[1]other = fibromyalgia, lupus, systemic lupus erythematosus, Wegeners granulomatosis, psoriasis, scleroderma, arthritis
[2]Basic education = primary and secondary level

For peer review only - http://bmjopen.bmj.com/site/about/guidelines.xhtml

Descriptive data on demographic and disease-related factors and fulfilment of physical activity recommendations of participants is displayed in Table 2.

Use of PA measures

Of the included 1,382 participants, 1,305 answered questions on PA measures. Of those, 600 (46%) reported that they used any kind of self-report or device-based measures to self-monitor PA. The most common used measure was the simple body worn device, used by 428 (33%), whereof 112 used it regularly. The complex body worn device were used by 182 (14%), whereof 56 used it regularly. One hundred and seven of 1,305 (8%) reported using digital or paper questionnaire and 104 (8%) used digital or paper diaries for self-monitoring of PA. Divided per country, the use of any kind of self-report or device-based measure differed between countries from 34% to 58% (Table 2).

Instructions of PA measures by HPs

Twenty-six of the 428 (6%) users of simple devices received instructions from HPs on how to use the device and of those 182 using the complex device, eight (4%) did. Health professionals instructed 52 (50%) of those 107 using digital/paper questionnaire and 10 out of 104 (10%) of those using diaries.

Perceived importance and confidence in self-monitoring of PA

All the 1,382 answered questions about importance and confidence. Most of the participants, 925 (67%) perceived it was important to measure PA and 558 (40%) reported they did not receive information about PA from any source. Of those receiving information about PA, 388 (28%) received the main source of information from a physiotherapist, 363 (26%) from a rheumatologist, 299 (22%) from internet, 159 (12%) from a general practitioner and 147 (11%)

For peer review only - http://bmjopen.bmj.com/site/about/guidelines.xhtml

from newspapers or magazines. A small number of the study participants received information from friends, 59 (4%), from an occupational therapist 46 (3%) or from a nurse 38 (3%).

The participants were most confident in using and interpreting PA data from simple body worn devices and paper questionnaires. They were least confident in using complex body worn sensors collecting multiple data, as well as using digital diaries (Table 3).

Table 3. Participants confidence/familiarity in using self-reports and devices based physical activity measures (n = 1305)

	n	Median (IQR)
Simple (0-10)*	1305	3 (8)
Complex sensor (0-10)	1305	1 (6)
Paper QR (0-10)	1305	3 (5)
Paper diary (0-10)	1304	1 (5)
Digital QR (0-10)	1305	1 (5)
Digital diary (0-10)	1305	0 (5)

* possible score range 0-10 0= not confident, 10= very confident
IQR= interquartile range, QR= questionnaire

Fulfilment of physical activity recommendations and its associated factors

A total of 503 of the 1,382 (36%) participants reported they fulfilled the recommendations of ≥ 150 minutes of PA on a moderate intensity per week, whereof 418 (39%) were female.

Univariate logistic regression analyses (Table 4) revealed that those who lived in Ireland (OR = 74.14, P< .001) and Sweden (OR = 1.68, P< .001) were more likely to fulfil the recommendations of PA as compared to those who lived in Denmark, whereas those who lived in Belgium (OR = 0.18, P< .001) were less likely to fulfil the recommendations of PA. The results also showed that participants who were <55 years of age (OR = 1.46, P= .001), working part- or fulltime (OR = 1.61, P< .001), being treated with non-biological disease modifying

For peer review only - http://bmjopen.bmj.com/site/about/guidelines.xhtml

medication (OR = 1.54, P< .001), with no perceived activity limitations in moderate activities (OR = 1.41, P< .001) and who were using a device to measure PA (OR = 1.93, P< .001) were more likely to fulfil the recommendations of PA. In contrast, those of male sex (OR = 0.68, P= .007) and not experiencing pain in the arm (OR = 0.56, P< .001) or leg (OR = 0.65, P< .001) were less likely to fulfil the recommendations of PA.

Table 4. Univariate and multiple logistic regression models for factors associated with fulfilment of recommendations of physical activity.

Variable	*Univariate regression*		*Multiple regression*	
	OR (95% CI)	*P-value*	OR (95% CI)	*P-value*
Country				
Belgium	0.18 (0.14–0.24)	**<.001**	0.21 (0.15–0.29)	**<.001**
Ireland	74.14 (10.22–537.52)	**<.001**	84.89 (11.64–619.23)	**<.001**
Sweden	1.68 (1.13–2.50)	**<.001**	1.68 (1.10–2.56)	**.017**
Denmark	1.00 (reference)		1.00 (reference)	
Age				
<55 years	1.46 (1.74–1.83)	**.001**	1.28 (0.97–1.68)	.076
≥55 years	1.00 (reference)		1.00 (reference)	
Sex				
Male	0.68 (0.51–0.90)	**.007**	0.99 (0.70–1.42)	.971
Female	1.00 (reference)		1.00 (reference)	
Work status				
Working	1.61 (1.28–2.01)	**<.001**	1.41 (0.87–1.50)	.565
Not working	1.00 (reference)		1.00 (reference)	
Education				
Higher education[1]	0.89 (0.81–1.27)	.895		
Basic education[2]	1.00 (reference)			
Bio-DMARDs medication				
Treatment	1.24 (0.97–1.60)	.084		
No treatment	1.00 (reference)			
Non-biological medication				
Treatment	1.54 (1.22–1.94)	**<.001**	0.92 (0.70–1.22)	.565
No treatment	1.00 (reference)		1.00 (reference)	
Activity limitation – moderate activities				
No limitation	1.41 (1.09–1.83)	**<.001**	1.92 (1.38–2.66)	**<.001**
Limitations	1.00 (reference)		1.00 (reference)	
Activity limitation – climbing stairs				

For peer review only - http://bmjopen.bmj.com/site/about/guidelines.xhtml

No limitation	1.00 (0.79–1.27)	.990		
Limitations	1.00 (reference)			
Pain arm				
No pain	0.56 (0.44–0.69)	**<.001**	0.76 (0.57–1.01)	.059
Pain	1.00 (reference)		1.00 (reference)	
Pain hand				
No pain	0.89 (0.72–1.12)	.328		
Pain	1.00 (reference)			
Pain foot				
No pain	1.10 (0.88–1.37)	.407		
Pain	1.00 (reference)			
Pain leg				
No pain	0.65 (0.52–0.81)	**<.001**	1.24 (0.92–1.66)	.153
Pain	1.00 (reference)		1.00 (reference)	
Think it is important to monitor physical activity				
Yes	1.26 (0.99–1.61)	.058		
No	1.00 (reference)			
Use device to measure physical activity				
Yes	1.93 (1.53–2.42)	**<.001**	1.56 (1.20–2.026)	**<.001**
No	1.00 (reference)		1.00 (reference)	
OR: odds ratio, [1]Higher education = university level, [2]Basic education = primary and secondary level				

The multivariable logistic regression model (Table 4) showed that participants who lived in Ireland (OR = 84.89, P< .001) and Sweden (OR = 1.68, P= .017) were more likely to fulfil the recommendations of PA as compared to those who lived in Denmark, whereas those who lived in Belgium (OR = 0.21, P< .001) were less likely to fulfil the recommendations of PA . Furthermore, participants not perceiving activity limitations in moderate activities (OR = 1.92, P< .001), and using a device to measure PA (OR = 1.56, P< .001) remained independent associated factors of fulfilment of recommendations of PA. In this model, 72.5% of the outcome was correctly classified.

DISCUSSION

For peer review only - http://bmjopen.bmj.com/site/about/guidelines.xhtml

To the best of our knowledge this is the first study describing the use of self-report and/or device-based PA measures in individuals with IJD in a real-world situation, across four European countries and its association to fulfilment of PA recommendations.

Overall, close to 50% of the participants used any kind of self-report or device-based measures to self-monitor PA. Furthermore, in our cohort 37% used device-based measures, which is comparable to the general population at the current time (25). This is promising given the motivation for PA which comes with the use of devices and also their advantages of simplicity and accuracy (25, 26). A between country difference of 34% to 58% was observed. It can be considered obvious that younger participants to a larger extent would be those who use wearable trackers. This could be considered supported by the data from Sweden, which has the lowest amount of tracker users, and also is the country with the fewest participants under the age of 55 years. However, Belgium had the highest number of tracker users although the country did not have the youngest group. Hence, other explanations to the between country differences may be sought beyond the variables measured in this study.

Only 112 (8%) of the participants used simple body worn devices and 56 (4%) used complex body worn devices regularly. This is in line with previous interventions aiming to increase PA using wearable trackers in people with rheumatic- and musculoskeletal diseases, showing that adherence to use of trackers over longer periods of time is not maintained (25).

Although a majority (67%) of our sample perceived it was important to measure PA, 40% did not receive information from a HP on why it is important, which is similar to an earlier study in an arthritis population (21). Of those using simple and complex devices only 6% and 4% respectively received instructions on how to use the devices. On the other hand, a substantial

For peer review only - http://bmjopen.bmj.com/site/about/guidelines.xhtml

part, 50%, reported they were instructed how to use questionnaires to measure PA. One explanation to this could be that HPs are used to using traditional questionnaires in clinic and therefore might be more prone to inform their patients about them. Since our participants responded to our survey in 2015-2016, measurement of PA has most likely become more common in clinical practice as well as HPs knowledge about different methods available. One recent study suggest that HPs may be more experienced in using PA devices today (27). The factor most strongly associated with engaging in PA is getting recommendation from a HP and previous research has identified low HP knowledge about PA and exercise as a reason for not guiding patients with IJD in PA (28). Even though HPs are aware of benefits and barriers in relation to PA in IJD, promotion and advice is conflicted by HPs lack of certainty with respect to joint damage and fear of flare-up (29). Moreover, recent studies highlight further educational needs and development for HPs to support PA in this population (19, 30). While PA devices can give the user real-time feedback of daily steps or energy expenditure, patients with IJDs would most likely benefit from also adding HPs support providing them with tailored feed-back to motivate PA (1). In fact, better long-term adherence has been shown in interventions incorporating feedback and behavioural change techniques compared with activity devices alone (31).

The participants' confidence in using self-reports and devices was low. This highlights the importance of HPs communicating with patients on the importance of PA measurement devices to build confidence in their use and their role in changing behaviour to improve PA levels. With HPs increased experience of using device-based measures (27), there is a possibility that communication to patients might increase.

For peer review only - http://bmjopen.bmj.com/site/about/guidelines.xhtml

Both uni- and multivariate regression models showed that participants living in Ireland and Sweden were more likely to fulfil the recommendations of PA as compared to the participants who lived in Denmark, whereas those in Belgium were less likely to fulfil the PA recommendation. This is in line with other studies describing PA levels in these countries (32-34). Furthermore, using a PA device was in addition an independent associated factor of adherence to PA recommendations, also when considering sociodemographic- and disease-related factors, support from HPs, and participants own perception of familiarity and confidence of using PA devices. As this is a cross-sectional study, we cannot determine if the measure itself motivates to PA in this sample, or if the more physically active participants use the measures for self-monitoring of their behaviour. However, using PA devices can be a valuable way to support people with IJDs to improve motivation or PA levels, and it is suggested to be taken into account in the promotion of PA (25, 35, 36). Not perceiving activity limitations was also a factor associated to adherence to PA recommendations in our sample . This corresponds well with a previous review by Veldhuijzen van Zanten et al, where activity limitation is described as a barrier of PA (1). These factors provide information for clinicians to identify which patients need more targeted support to improve their PA levels, an important consideration in managing busy clinical loads.

Limitations

First, the study had a cross-sectional design therefore, conclusions on causal relationships cannot be drawn. This could have been helped by a follow up over time to allow for firmer conclusions about causality.

Second, the questionnaire was based on self-report through social networking, which may have attracted socially desired answers. This is an inherent limitation in most surveys and while it was ensured the data collection was anonymous, self-reports are never free from bias

For peer review only - http://bmjopen.bmj.com/site/about/guidelines.xhtml

(37). Moreover, most of those who answered were those involved in the patient organization, probably with a specific interest in their arthritis, and most likely had a particular interest in the topic. However, as inclusion also took place through outpatient clinics in both Belgium and Sweden, our sample is probably representative of a broader range of characteristics as the patient organizations' members are generally older, thus not working and not on disability leave as they are retired. It should be noted that the overall response rate could not be calculated, as exact numbers of members of the patient organizations were not available or could not be retrieved. Another point which could be regarded as a limitation is that the recruitment procedure through the patient organizations was not similar in each country. In Belgium, Denmark and Ireland invitations to participate were sent via email to members of the patient organizations while in Sweden recruitment took place through the Swedish Rheumatism Association's Facebook page. Furthermore, PA interventions using wearable trackers in people with IJDs have increased the past years (38, 39). This may have promoted increased awareness and use of PA devices since our data was collected. However, we are not aware of other studies which have addressed the same issues as we have. Our data should therefore remain relevant as a starting point for further studies.

As reported earlier in our previous study, investigating rheumatology HPs awareness of PA measures in people with IJDs (30) we cannot neglect the risk of respondents having misinterpreted questions in the survey. Simultaneously with the survey used in the prior study (30) the present survey was designed in English and translated into Swedish, Danish, French and Flemish with results back-translated into English for the reporting. Through the different translations, there is a risk that valuable information was lost. Face validity was investigated in each country before the survey was used, and part of the included questions were other validated questionnaires (23, 40).

For peer review only - http://bmjopen.bmj.com/site/about/guidelines.xhtml

CONCLUSION

Despite these limitations, this study contributes and provides insight into the use of PA measures and devices in a real-world setting by people with IJDs. This is important in order to enhance the collaborative promotion of PA between health professional and patient (31). The study found that almost half of the participants with IJDs used self- or deviced based PA measures, signaling the value of such devices in improving overall PA awareness and engagement. Moreover, participants meeting public PA health guidelines were engaged in self-monitoring PA. The study also identified factors associated with PA device use allowing for more targeted management of people with IJDs to improve PA. It also highlights the need for HP engagement with PA advice and understanding of PA devices as an important part of the care of people with IJDs.

Acknowledgement

The authors thank Sara Wiljeminis who assisted in designing the survey and collecting the data.

Contributor ship statement

NB, BN, NK, BAE, and TWS were the original authors who submitted the grant, designed the study, oversaw the data collection and analysis in their countries, prepared the paper and read and commented on all drafts and agreed on the final manuscript. SGMcK, and NMH were research assistants who assisted with designing the survey, collecting the data, and commented on manuscript drafts. DMC and PH undertook the statistical analyses and commented on all manuscript drafts.

Competing interests

None declared

Funding

For peer review only - http://bmjopen.bmj.com/site/about/guidelines.xhtml

The study was funded by the European Leauge Against Rheumatism Health Professional Research Grant 2015.

Data Sharing Statement

No additional data available

Ethics Approval

This study was approved by the Medical Ethical Committee of the University Hospitals Leuven S58849 in Belgium, by the Ethics Committee of the Capital Region of Denmark H-15013386 in Denmark, by the University of Limerick research ethics committee: 2015_09_02_EHS in Ireland and by the Swedish Etikprövningsmyndigheten 2015/1785-31/5 in Sweden.

REFERENCES

1. Veldhuijzen van Zanten JJ, Rouse PC, Hale ED, Ntoumanis N, Metsios GS, Duda JL, et al. Perceived Barriers, Facilitators and Benefits for Regular Physical Activity and Exercise in Patients with Rheumatoid Arthritis: A Review of the Literature. Sports Med. 2015;45(10):1401-12.

2. Collado-Mateo D, Lavín-Pérez AM, Peñacoba C, Del Coso J, Leyton-Román M, Luque-Casado A, et al. Key Factors Associated with Adherence to Physical Exercise in Patients with Chronic Diseases and Older Adults: An Umbrella Review. Int J Environ Res Public Health. 2021;18(4).

3. Caspersen CJ, Powell KE, Christenson GM. Physical activity, exercise, and physical fitness: definitions and distinctions for health-related research. Public health reports (Washington, DC : 1974). 1985;100(2):126-31.

For peer review only - http://bmjopen.bmj.com/site/about/guidelines.xhtml

4. Durcan L, Wilson F, Cunnane G. The effect of exercise on sleep and fatigue in rheumatoid arthritis: a randomized controlled study. The Journal of rheumatology. 2014;41(10):1966-73.

5. Rausch Osthoff AK, Niedermann K, Braun J, Adams J, Brodin N, Dagfinrud H, et al. 2018 EULAR recommendations for physical activity in people with inflammatory arthritis and osteoarthritis. Ann Rheum Dis. 2018;77(9):1251-60.

6. Hootman JM, Macera CA, Ham SA, Helmick CG, Sniezek JE. Physical activity levels among the general US adult population and in adults with and without arthritis. Arthritis Rheum. 2003;49(1):129-35.

7. Tierney M, Fraser A, Kennedy N. Physical activity in rheumatoid arthritis: a systematic review. J Phys Act Health. 2012;9(7):1036-48.

8. Jacquemin C, Servy H, Molto A, Sellam J, Foltz V, Gandjbakhch F, et al. Physical Activity Assessment Using an Activity Tracker in Patients with Rheumatoid Arthritis and Axial Spondyloarthritis: Prospective Observational Study. JMIR mHealth and uHealth. 2018;6(1):e1.

9. Barker J, Smith Byrne K, Doherty A, Foster C, Rahimi K, Ramakrishnan R, et al. Physical activity of UK adults with chronic disease: cross-sectional analysis of accelerometer-measured physical activity in 96 706 UK Biobank participants. International journal of epidemiology. 2019;48(4):1167-74.

10. Haider S, Sedlak M, Kapan A, Grabovac I, Lamprecht T, Erlacher L, et al. Factors Associated with Objectively Measured Physical Activity in Patients with Seropositive Rheumatoid Arthritis. Int J Environ Res Public Health. 2020;17(23).

11. Michie S, Ashford S, Sniehotta FF, Dombrowski SU, Bishop A, French DP. A refined taxonomy of behaviour change techniques to help people change their physical activity and healthy eating behaviours: the CALO-RE taxonomy. Psychology & health. 2011;26(11):1479-98.

For peer review only

For peer review only - http://bmjopen.bmj.com/site/about/guidelines.xhtml

12. Page EJ, Massey AS, Prado-Romero PN, Albadawi S. The Use of Self-Monitoring and Technology to Increase Physical Activity: A Review of the Literature. Perspectives on behavior science. 2020;43(3):501-14.

13. Compernolle S, DeSmet A, Poppe L, Crombez G, De Bourdeaudhuij I, Cardon G, et al. Effectiveness of interventions using self-monitoring to reduce sedentary behavior in adults: a systematic review and meta-analysis. The international journal of behavioral nutrition and physical activity. 2019;16(1):63.

14. Patel MS, Asch DA, Volpp KG. Wearable devices as facilitators, not drivers, of health behavior change. Jama. 2015;313(5):459-60.

15. Dowd KP, Szeklicki R, Minetto MA, Murphy MH, Polito A, Ghigo E, et al. A systematic literature review of reviews on techniques for physical activity measurement in adults: a DEDIPAC study. The international journal of behavioral nutrition and physical activity. 2018;15(1):15.

16. Larkin L, Nordgren B, Purtill H, Brand C, Fraser A, Kennedy N. Criterion Validity of the activPAL Activity Monitor for Sedentary and Physical Activity Patterns in People Who Have Rheumatoid Arthritis. Phys Ther. 2016;96(7):1093-101.

17. Arends S, Hofman M, Kamsma YP, van der Veer E, Houtman PM, Kallenberg CG, et al. Daily physical activity in ankylosing spondylitis: validity and reliability of the IPAQ and SQUASH and the relation with clinical assessments. Arthritis Res Ther. 2013;15(4):R99.

18. Smith RD, Dziedzic KS, Quicke JG, Holden MA, McHugh GA, Healey EL. Identification and Evaluation of Self-Report Physical Activity Instruments in Adults With Osteoarthritis: A Systematic Review. Arthritis Care Res (Hoboken). 2019;71(2):237-51.

19. Swärdh E, Opava C, Brodin N. Physical activity in patients with rheumatoid arthritis - an agile lifelong behaviour: a qualitative meta-synthesis. RMD open. 2021;7(2).

For peer review only - http://bmjopen.bmj.com/site/about/guidelines.xhtml

20. Freid LM, Ogdie A, Baker JF. Physical Activity Patterns in People With Inflammatory Arthritis Indicate They Have not Received Recommendation-Based Guidance From Health Care Providers. ACR open rheumatology. 2020;2(10):582-7.

21. Fontaine KR, Bartlett SJ, Heo M. Are health care professionals advising adults with arthritis to become more physically active? Arthritis Rheum. 2005;53(2):279-83.

22. Vandenbroucke JP, von Elm E, Altman DG, Gøtzsche PC, Mulrow CD, Pocock SJ, et al. Strengthening the Reporting of Observational Studies in Epidemiology (STROBE): explanation and elaboration. PLoS medicine. 2007;4(10):e297.

23. Wendel-Vos GC, Schuit AJ, Saris WH, Kromhout D. Reproducibility and relative validity of the short questionnaire to assess health-enhancing physical activity. Journal of clinical epidemiology. 2003;56(12):1163-9.

24. Ainsworth BE, Haskell WL, Leon AS, Jacobs DR, Jr., Montoye HJ, Sallis JF, et al. Compendium of physical activities: classification of energy costs of human physical activities. Medicine and science in sports and exercise. 1993;25(1):71-80.

25. Davergne T, Pallot A, Dechartres A, Fautrel B, Gossec L. Use of Wearable Activity Trackers to Improve Physical Activity Behavior in Patients With Rheumatic and Musculoskeletal Diseases: A Systematic Review and Meta-Analysis. Arthritis Care Res (Hoboken). 2019;71(6):758-67.

26. Sylvia LG, Bernstein EE, Hubbard JL, Keating L, Anderson EJ. Practical guide to measuring physical activity. Journal of the Academy of Nutrition and Dietetics. 2014;114(2):199-208.

27. Leese J, Macdonald GG, Tran BC, Wong R, Backman CL, Townsend AF, et al. Using Physical Activity Trackers in Arthritis Self-Management: A Qualitative Study of Patient and Rehabilitation Professional Perspectives. Arthritis Care Res (Hoboken). 2019;71(2):227-36.

For peer review only - http://bmjopen.bmj.com/site/about/guidelines.xhtml

28. O'Donnell S, Rusu C, Bernatsky S, Hawker G, Canizares M, Mackay C, et al. Exercise/physical activity and weight management efforts in Canadians with self-reported arthritis. Arthritis Care Res (Hoboken). 2013;65(12):2015-23.

29. Halls S, Law RJ, Jones JG, Markland DA, Maddison PJ, Thom JM. Health Professionals' Perceptions of the Effects of Exercise on Joint Health in Rheumatoid Arthritis Patients. Musculoskeletal care. 2017;15(3):196-209.

30. Kennedy NM, McKenna SG, O'Neill A, Appel Esbensen B, Swinnen TW, Nordgren B, et al. A survey across four European countries to determine rheumatology health professionals' awareness of physical activity measures in people with inflammatory joint diseases. BMJ open. 2018;8(5):e020809.

31. Brickwood KJ, Watson G, O'Brien J, Williams AD. Consumer-Based Wearable Activity Trackers Increase Physical Activity Participation: Systematic Review and Meta-Analysis. JMIR mHealth and uHealth. 2019;7(4):e11819.

32. McKenna S, Tierney M, O'Neill A, Fraser A, Kennedy N. Sleep and physical activity: a cross-sectional objective profile of people with rheumatoid arthritis. Rheumatol Int. 2018;38(5):845-53.

33. Bremander A, Malm K, Andersson ML. Physical activity in established rheumatoid arthritis and variables associated with maintenance of physical activity over a seven-year period - a longitudinal observational study. BMC rheumatology. 2020;4:53.

34. Rasmussen JO, Primdahl J, Fick W, Bremander A. Physical activity in people with axial spondyloarthritis and the impact of overall attitudes, barriers, and facilitators: A cross-sectional study. Musculoskeletal care. 2020;18(4):510-8.

35. Li C, Chen X, Bi X. Wearable activity trackers for promoting physical activity: A systematic meta-analytic review. International journal of medical informatics. 2021;152:104487.

For peer review only

For peer review only - http://bmjopen.bmj.com/site/about/guidelines.xhtml

36. Ashur C, Cascino TM, Lewis C, Townsend W, Sen A, Pekmezi D, et al. Do Wearable Activity Trackers Increase Physical Activity Among Cardiac Rehabilitation Participants? A SYSTEMATIC REVIEW AND META-ANALYSIS. Journal of cardiopulmonary rehabilitation and prevention. 2021;41(4):249-56.

37. van de Mortel TF. Faking it: social desirability response bias in self-report research. Australian Journal of Advanced Nursing. 2008;25(4):40-8.

38. Davergne T, Rakotozafiarison A, Servy H, Gossec L. Wearable Activity Trackers in the Management of Rheumatic Diseases: Where Are We in 2020? Sensors (Basel, Switzerland). 2020;20(17).

39. Pellegrini CA, Powell SM, Mook N, DeVivo K, Ehrlich-Jones L. Use of Physical Activity Monitors in Rheumatic Populations. Current rheumatology reports. 2018;20(12):73.

40. Linton SJ, Halldén K. Can we screen for problematic back pain? A screening questionnaire for predicting outcome in acute and subacute back pain. The Clinical journal of pain. 1998;14(3):209-15.

For peer review only - http://bmjopen.bmj.com/site/about/guidelines.xhtml

Supplementary file – Survey

A survey of people with inflammatory joint diseases use of physical activity measures

Demographics

* 1. Gender

○ Male

○ Female

* 2. What is your age?

○ 18 to 24

○ 25 to 34

○ 35 to 44

○ 45 to 54

○ 55 to 64

○ 65 to 74

○ 75 or older

* 3. What type of arthritis have you been diagnosed with?

○ Rheumatoid Arthritis (RA)

○ Ankylosing Spondylitis (AS)

○ Psoriatic Arthritis (PsA)

Other (please specify)

* 4. What year did you first experience your symptoms?

For peer review only

* 5. What body region do you have pain in?

- [ ] Arm
- [ ] Hand
- [ ] Foot
- [ ] Leg
- [ ] Spine
- [ ] Neck
- [ ] Head
- [ ] Other

Other (please specify)

6. Does your health limit you in the following activities?

	Yes, limited a lot	Yes, limited a little	No, not limited at all
Moderate activities e.g. moving a table, vacuum cleaning, bowling, golf etc.	☐	☐	☐
Climbing several flights of stairs	☐	☐	☐

For peer review only

* 7. What type of medication are you on?

- I do not take medications
- Biological DMARDS
- Non-biological DMARDS
- I do not know

Other (please specify)

* 8. What is your working situation?

- Full-time employment
- Part-time employment
- Unemployed
- Retired (due to age)
- Disability
- Housewife/Househusband

Other (please specify)

* 9. What is your highest level of education?

- Primary level
- Secondary level
- Third level

Other (please specify)

Views on measuring Physical Activity

* 10. Do you think it is important to measure physical activity?

- Yes
- No

11. Why do you/don't you think it is important to measure physical activity?

For peer review only

12. Where do you find your information on measuring physical activity?

- [ ] I don't
- [ ] Physiotherapist
- [ ] Rheumatologist
- [ ] Occupational Therapist
- [ ] Nurse
- [ ] Friends
- [ ] GP/Family Doctor
- [ ] Internet
- [ ] Magazine/Newspaper

Other (please specify)

Physical Activity Measurement - Objective measurement

Physical Activity is important for people with IJD's. While the measurement and monitoring of same can be a challenge, it has a number of essential uses. The following questions are concerned with the more popular ways in which Physical Activity is monitored and your answers will guide the evidence base and practice of health promotion.

13. How familiar are you with the following devices/ways in monitoring physical activity? (0 is not familiar and 10 is very familiar)

For peer review only

	0	1	2	3	4	5	6	7	8	9	10
PEDOMETER 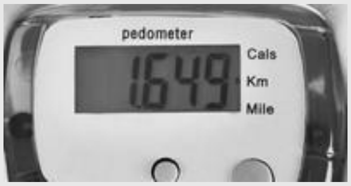	○	○	○	○	○	○	○	○	○	○	○
ACCELEROMETER 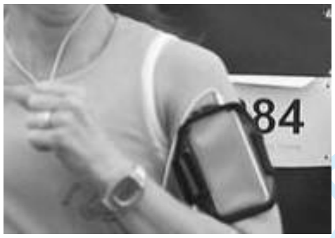	○	○	○	○	○	○	○	○	○	○	○
INTERNET 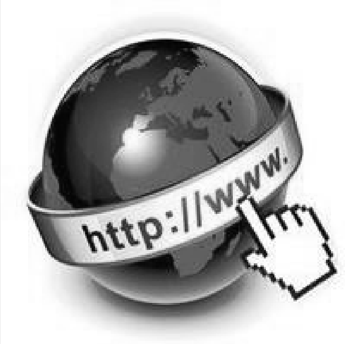	○	○	○	○	○	○	○	○	○	○	○
SMARTPHONE APP 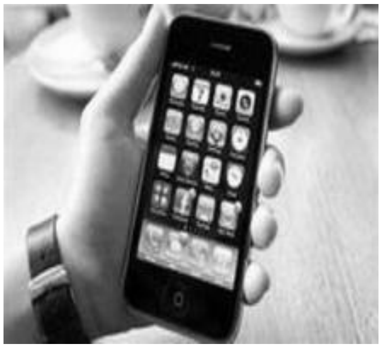	○	○	○	○	○	○	○	○	○	○	○

For peer review only

	0	1	2	3	4	5	6	7	8	9	10
QUESTIONNAIRE	○	○	○	○	○	○	○	○	○	○	○
DIARY	○	○	○	○	○	○	○	○	○	○	○

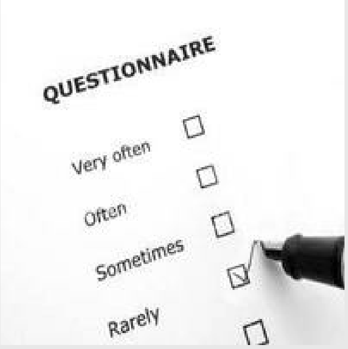

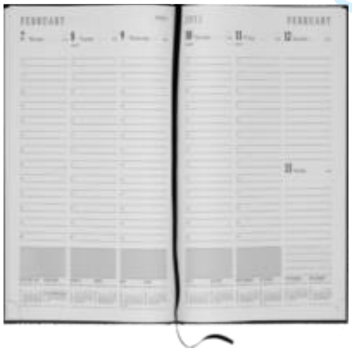

For peer review only

14. The following questions are in relation to PEDOMETERS

How familiar are you in using a Pedometer to measure Physical Activity?

- ○ Very familiar
- ○ Somewhat familiar
- ○ Vaguely familiar
- ○ Not familiar

15. Do you own a Pedometer?

- ○ Yes
- ○ No

16. If Yes, where did you get the Pedometer?

- I bought it
- A gift
- Provided by my employer/work place
- From a study
- From a Health Professional

Other (please specify)

17. If Yes, how frequently do you wear a Pedometer?

- Every Day
- Most Days
- Rarely wear it
- Never wear it

18. Have you been instructed in how to use a Pedometer by a Health Professional within Rheumatology?

- Yes
- No

19. If Yes, who has instructed you in using it?

- Nurse
- Rheumatologist
- Physiotherapist
- Occupational Therapist

Other (please specify)

For peer review only

20. How familiar are you in reading the results from a Pedometer?

- ◯ Very familiar
- ◯ Somewhat familiar
- ◯ Vaguely familiar
- ◯ Not familiar

21. The following questions are in relation to ACCELEROMETERS

How familiar are you in using an Accelerometer for measuring Physical Activity?

- ◯ Very familiar
- ◯ Somewhat familiar
- ◯ Vaguely familiar
- ◯ Not familiar

22. Do you own an Accelerometer?

- ◯ Yes
- ◯ No

23. If Yes, where did you get the Accelerometer?

- ◯ I bought it
- ◯ A gift
- ◯ Provided by my employer/work place
- ◯ From a study
- ◯ From a Health Professional

Other (please specify)

For peer review only

24. If Yes, how frequently do you wear an Accelerometer?

- Every Day
- Most Days
- Rarely wear it
- Never wear it

25. Have you been instructed in how to use an Accelerometer by a Health Professional within Rheumatology?

- Yes
- No

26. If Yes, who has instructed you in using it?

- Nurse
- Rheumatologist
- Physiotherapist
- Occupational Therapist

Other (please specify)

27. How familiar are you in reading the results from an Accelerometer?

- Very familiar
- Somewhat familiar
- Vaguely familiar
- Not familiar

28. The following questions are in relation to the INTERNET

How familiar are you in using the INTERNET for measuring Physical Activity?

- Very familiar
- Somewhat familiar
- Vaguely familiar
- Not familiar

For peer review only

29. Do you use the INTERNET for measuring Physical Activity?

- ◯ Yes
- ◯ No

30. If Yes, have any of the following recommended you to use it?

- ◯ Nurse
- ◯ Rheumatologist
- ◯ Physiotherapist
- ◯ Occupational Therapist

Other (please specify)

31. If Yes, how frequently do you use the INTERNET to measure your Physical Activity?

- ◯ Every Day
- ◯ Most Days
- ◯ Rarely wear it
- ◯ Never wear it

32. Have you been instructed in how to use the INTERENET to measure your Physical Activity by a Health Professional within Rheumatology?

- ◯ Yes
- ◯ No

33. If Yes, who has instructed you in using it?

- ◯ Nurse
- ◯ Rheumatologist
- ◯ Physiotherapist
- ◯ Occupational Therapist

Other (please specify)

For peer review only

34. How familiar are you in reading the results of your Physical Activity from the INTERNET?

- ◯ Very familiar
- ◯ Somewhat familiar
- ◯ Vaguely familiar
- ◯ Not familiar

35. The following questions are in relation to SMARTPHONE APPS

How familiar are you in using a Smartphone App for measuring Physical Activity?

- ◯ Very familiar
- ◯ Somewhat familiar
- ◯ Vaguely familiar
- ◯ Not familiar

36. Do you have a Smartphone App for Physical Activity?

- ◯ Yes
- ◯ No

For peer review only

37. If Yes, where did you get your Smartphone App?

- I downloaded it - Free
- I downloaded it - Paid
- A gift
- Provided by my employer/work place
- From a study
- From a Health Professional

Other (please specify)

38. If Yes, how frequently do you use a Smartphone App?

- Every Day
- Most Days
- Rarely wear it
- Never wear it

39. Have you been instructed in how to use a Smartphone App by a Health Professional within Rheumatology?

- Yes
- No

40. If Yes, who has instructed you in using it?

- Nurse
- Rheumatologist
- Physiotherapist
- Occupational Therapist

Other (please specify)

For peer review only

41. How familiar are you in reading the results from a Smartphone App?

- Very familiar
- Somewhat familiar
- Vaguely familiar
- Not familiar

42. The following questions are in relation to QUESTIONNAIRES

How familiar are you in using a Questionnaire or Diary for measuring Physical Activity?

- Very familiar
- Somewhat familiar
- Vaguely familiar
- Not familiar

43. Do you use a Questionnaire for measuring Physical Activity?

- Yes
- No

44. If Yes, have any of the following recommended you to use it?

- Nurse
- Rheumatologist
- Physiotherapist
- Occupational Therapist

Other (please specify)

For peer review only

45. If Yes, how frequently do you use a Questionnaire to measure your Physical Activity?

- Every Day
- Most Days
- Rarely wear it
- Never wear it

46. How familiar are you in reading the results of your Physical Activity from a Questionnaire?

- Very familiar
- Somewhat familiar
- Vaguely familiar
- Not familiar

47. The following questions are in relation to DIARIES

How familiar are you in using a Diary for measuring Physical Activity?

- Very familiar
- Somewhat familiar
- Vaguely familiar
- Not familiar

48. Do you use a Diary for measuring Physical Activity?

- Yes
- No

49. If Yes, have any of the following recommended you to use it?

- Nurse
- Rheumatologist
- Physiotherapist
- Occupational Therapist

Other (please specify)

For peer review only

50. If Yes, how frequently do you use a Diary to measure your Physical Activity?

- Every Day
- Most Days
- Rarely wear it
- Never wear it

51. How familiar are you in reading the results of your Physical Activity from a Diary?

- Very familiar
- Somewhat familiar
- Vaguely familiar
- Not familiar

Barriers and Preferences

52. Do you think there are any barriers to people with arthritis measuring their own physical activity levels?

- Yes
- No

53. If Yes, please give an example

54. In your opinion, what is the best way to measure Physical Activity for people with arthritis?

* Pedometer
* Accelerometer
* Internet
* Smartphone app
* Questionnaire
* Diary

For peer review only

Current Physical Activity Levels

* 55. Think about an average week in the past few months and please answer the following regarding yourown Physical activites

	How many days per week?	Average time in minutes per day?	Intensity of activity
Commuting Activities i.e. Walk, Cycle etc.			
Work Activities i.e. Walking, Standing, Lifting etc.			
Leisure Time Activities i.e. Walking, Cycling, Gardening etc.			
Sport Activities i.e. Tennis, Football, Rugby etc.			
Household Activities i.e. Cleaning, Washing etc.			

For peer review only

STROBE 2007 (v4) Statement—Checklist of items that should be included in reports of *cross-sectional studies*

Section/Topic	Item #	Recommendation	Reported on page #
**Title and abstract**	1	(*a*) Indicate the study's design with a commonly used term in the title or the abstract	1-3
		(*b*) Provide in the abstract an informative and balanced summary of what was done and what was found	2
**Introduction**			
Background/rationale	2	Explain the scientific background and rationale for the investigation being reported	3-4
Objectives	3	State specific objectives, including any prespecified hypotheses	4
**Methods**			
Study design	4	Present key elements of study design early in the paper	5
Setting	5	Describe the setting, locations, and relevant dates, including periods of recruitment, exposure, follow-up, and data collection	5-7
Participants	6	(*a*) Give the eligibility criteria, and the sources and methods of selection of participants	5
Variables	7	Clearly define all outcomes, exposures, predictors, potential confounders, and effect modifiers. Give diagnostic criteria, if applicable	5-6
Data sources/ measurement	8*	For each variable of interest, give sources of data and details of methods of assessment (measurement). Describe comparability of assessment methods if there is more than one group	5-8
Bias	9	Describe any efforts to address potential sources of bias	Supplementary file
Study size	10	Explain how the study size was arrived at	-
Quantitative variables	11	Explain how quantitative variables were handled in the analyses. If applicable, describe which groupings were chosen and why	8
Statistical methods	12	(*a*) Describe all statistical methods, including those used to control for confounding	8
		(*b*) Describe any methods used to examine subgroups and interactions	8
		(*c*) Explain how missing data were addressed	-
		(*d*) If applicable, describe analytical methods taking account of sampling strategy	-
		(*e*) Describe any sensitivity analyses	-
**Results**			

For peer review only

Participants	13*	(a) Report numbers of individuals at each stage of study—eg numbers potentially eligible, examined for eligibility, confirmed eligible, included in the study, completing follow-up, and analysed	8-9
		(b) Give reasons for non-participation at each stage	-
		(c) Consider use of a flow diagram	-
Descriptive data	14*	(a) Give characteristics of study participants (eg demographic, clinical, social) and information on exposures and potential confounders	Table 2
		(b) Indicate number of participants with missing data for each variable of interest	8-9, Table 2
Outcome data	15*	Report numbers of outcome events or summary measures	
Main results	16	(*a*) Give unadjusted estimates and, if applicable, confounder-adjusted estimates and their precision (eg, 95% confidence interval). Make clear which confounders were adjusted for and why they were included	Table 5
		(*b*) Report category boundaries when continuous variables were categorized	Table 1
		(*c*) If relevant, consider translating estimates of relative risk into absolute risk for a meaningful time period	Not applicable
Other analyses	17	Report other analyses done—eg analyses of subgroups and interactions, and sensitivity analyses	-
**Discussion**			
Key results	18	Summarise key results with reference to study objectives	10-11
Limitations	19	Discuss limitations of the study, taking into account sources of potential bias or imprecision. Discuss both direction and magnitude of any potential bias	13
Interpretation	20	Give a cautious overall interpretation of results considering objectives, limitations, multiplicity of analyses, results from similar studies, and other relevant evidence	10-12 + 14
Generalisability	21	Discuss the generalisability (external validity) of the study results	12, 13
**Other information**			
Funding	22	Give the source of funding and the role of the funders for the present study and, if applicable, for the original study on which the present article is based	15

*Give information separately for cases and controls in case-control studies and, if applicable, for exposed and unexposed groups in cohort and cross-sectional studies.

Note: An Explanation and Elaboration article discusses each checklist item and gives methodological background and published examples of transparent reporting. The STROBE checklist is best used in conjunction with this article (freely available on the Web sites of PLoS Medicine at http://www.plosmedicine.org/, Annals of Internal Medicine at http://www.annals.org/, and Epidemiology at http://www.epidem.com/). Information on the STROBE Initiative is available at www.strobe-statement.org.

For peer review only
